# DNA Methylation of the Autonomous Pathway Is Associated with Flowering Time Variations in *Arabidopsis thaliana*

**DOI:** 10.3390/ijms25137478

**Published:** 2024-07-08

**Authors:** Hongjie Xie, Xinchen Li, Yuli Sun, Lei Lin, Keke Xu, Huan Lu, Biao Cheng, Siming Xue, Dan Cheng, Sheng Qiang

**Affiliations:** Weed Research Laboratory, College of Life Science, Nanjing Agricultural University, Nanjing 210095, China; xhj@njau.edu.cn (H.X.); lixinchen@stu.njau.edu.cn (X.L.); sunyuli@tal.com (Y.S.); 2021116084@stu.njau.edu.cn (L.L.); 2018116018@stu.njau.edu.cn (K.X.); huan.lu@njau.edu.cn (H.L.); 18751966212@139.com (B.C.); 13643829050@163.com (S.X.); softcheng@njau.edu.cn (D.C.)

**Keywords:** codon degeneracy substitution, gene expression, transcription factor, *FVE* gene

## Abstract

Plant flowering time is affected by endogenous and exogenous factors, but its variation patterns among different populations of a species has not been fully established. In this study, 27 *Arabidopsis thaliana* accessions were used to investigate the relationship between autonomous pathway gene methylation, gene expression and flowering time variation. DNA methylation analysis, RT-qPCR and transgenic verification showed that variation in the flowering time among the *Arabidopsis* populations ranged from 19 to 55 days and was significantly correlated with methylation of the coding regions of six upstream genes in the autonomous pathway, *FLOWERING LOCUS VE* (*FVE*), *FLOWERING LOCUS Y* (*FY*), *FLOWERING LOCUS D* (*FLD*), *PEPPER* (*PEP*), *HISTONE DEACETYLASE 5* (*HAD5*) and *Pre-mRNA Processing Protein 39-1* (*PRP39-1*), as well as their relative expression levels. The expression of *FVE* and *FVE*(*CS*) was modified separately through degenerate codon substitution of cytosine and led to earlier flowering of transgenic plants by 8 days and 25 days, respectively. An accurate determination of methylated sites in *FVE* and *FVE*(*CS*) among those transgenic plants and the recipient Col-0 verified the close relationship between the number of methylation sites, expression and flowering time. Our findings suggest that the methylation variation of these six key upstream transcription factors was associated with the gene expression level of the autonomous pathway and flowering time in Arabidopsis. The *FVE*(*CS*) and *FVE* genes in transgenic plants tended to be hypermethylated, which could be a protective mechanism for plants. However, modification of gene sequences through degenerate codon substitution to reduce cytosine can avoid hypermethylated transferred genes in transgenic plants. It may be possible to partially regulate the flowering of plants by modified trans-epigenetic technology.

## 1. Introduction

Flowering is the most important life-history trait of plants, representing a fundamental transition in development from vegetative growth to reproductive growth. Plants have evolved a complex network of regulatory pathways to sense and integrate exogenous and endogenous signals to precisely control flowering time [1]. Flowering time varies significantly among plant species, ranging from a few days to years [2]. Flowering time can also differ greatly within a species. *Arabidopsis thaliana* (*A. thaliana* henceforth) grows throughout the Northern Hemisphere, and wild accessions show extensive flowering time variation [3,4]. The extensive variation in flowering time may contribute to the survival range of this species. Many factors may lead to differences in flowering time in *A. thaliana*, such as differential expression of flowering-related genes and biotic or abiotic factors [5,6,7,8,9,10].

**Figure 1:**
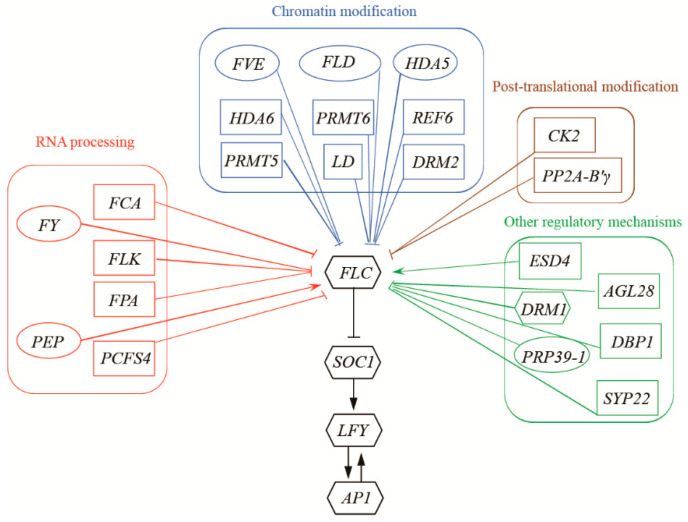
Methylation levels of gene coding regions in *A. thaliana* autonomous pathway. Ellipses: genes with methylation related to flowering time and leaf number; Rectangles: gene coding regions universally methylated in the 27 *A. thaliana* accessions but not related to flowering time or leaf number; and Hexagons: gene coding regions generally not methylated in 27 *A. thaliana* accessions and not related to flowering time or leaf number.

The flowering time of *A. thaliana* has been extensively studied, and eight pathways have been found to participate in flowering time regulation, including pathways related to the photoperiod, vernalization, temperature, gibberellins, autonomy, age, the circadian clock and the sugar budget [9,11,12]. Among these pathways, the autonomous pathway controls the flowering of *A. thaliana* through the complicated regulation of FLOWERING LOCUS C (FLC), involving mainly RNA processing, chromatin modification, post-translational modification and other mechanisms [7,13] (Figure 1). An autonomous pathway complex (AuPC) containing FLOWERING LOCUS D (FLD), LUMINIDEPENDENS (LD) and SET DOMAIN GROUP 26 (SDG26) suppresses the *FLC* expression partly through histone modifications [14]. The SDG26 also regulates the *FLC* expression and flowering time independently [14,15]. 5-Azacytidine (5-azaC), a potent inhibitor of DNA methyltransferases, offers a straightforward method for experimentally altering the DNA methylation landscape in plants. Utilizing 5-azaC has been critical for elucidating the complexities of DNA methylation patterns, gene regulatory mechanisms and epigenetic reprogramming. Previous studies with 5-azaC-treated [16], *ddm1*-mutant or *MET1* antisense plants [17] have revealed the influence of methylation on the transcription of certain genes in the autonomous pathway by changing the plant’s DNA methylation status at the genomic level. Tools targeting the removal of DNA methylation at specific loci in the genome with high specificity have also been established [18]. Based on these techniques, a stable *fwa* epiallele consisting of the loss of DNA methylation in the promoter of the *FLOWERING WAGENINGEN* (*FWA*) gene was obtained and well characterized [18,19]. However, the functions of DNA methylation of specific genes involved in flowering time variation still need to be further illustrated.

DNA methylation is associated with various processes, such as transposon silencing, gene expression and stress responses [20,21,22,23,24]. Although DNA methylation has been extensively studied, most of the studies related to DNA methylation function have been based on epigenomic analyses [20,25]; the effects of DNA methylation of a single gene still need further study. Our previous studies showed that the methylation of the *ICE1* gene may play an important role in the cold tolerance evolution of the invasive plant species *Ageratina adenophora* (crofton weed), weedy rice (*Oryza sativa* L. f. *spontanea*) and *A. thaliana* [24,26,27]. Some studies have also investigated the role of DNA methylation in the floral regulation of *A. thaliana* [17,28]. However, conclusions regarding the function of cytosine methylation of gene coding regions in plants are inconsistent [20,21,29,30,31,32,33]. Therefore, in this study, epigenomic analysis was performed to establish correlations between the methylation and expression levels of specific genes in the autonomous pathway and the flowering time of *A. thaliana*, after which the identified methylated gene was transformed to verify the effects of its methylation level. The main objective was to investigate the effects of methylation of specific genes involved in the autonomous pathway on flowering time variation in *A. thaliana*.

## 2. Results

### 2.1. Relationship between Flowering Time and the Methylation of Autonomous Pathway Genes

**Figure 2:**
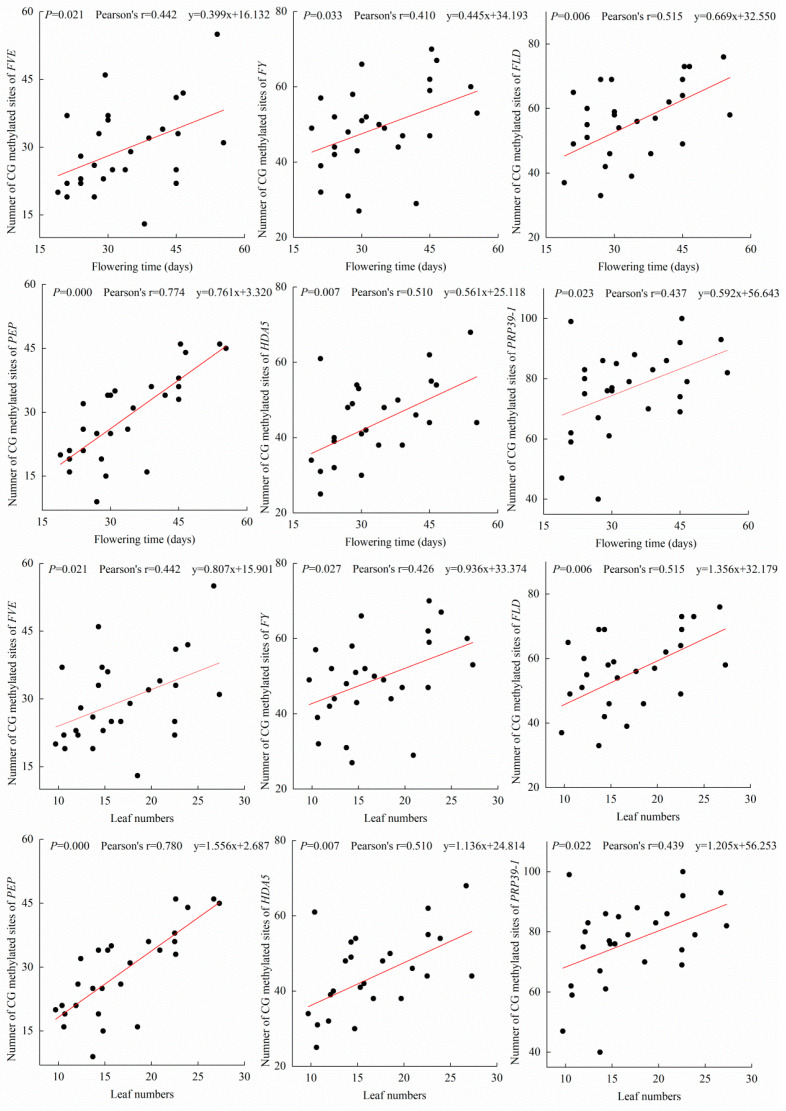
Correlation plot of methylation levels of the coding regions of *A. thaliana* autonomous pathway genes, flowering time and leaf number. mC: total number of methylated sites of coding regions.

The 27 *A. thaliana* accessions displayed various flowering times ranging from 19–55.4 days, which corresponded to different total numbers of leaves after flowering (Table S1). Methylomic analysis revealed no significant variations (*p* > 0.05) in CG, CHG, and CHH methylation of protein-coding genes across most *A. thaliana* accessions, with the exception of CG methylation in Cvi-0 and Bs-1 (Figure S1). However, methylomic analysis revealed that 27 genes in the *A. thaliana* autonomous pathway were differentially methylated, with decreasing methylation of the upstream genes to the downstream genes, with the exception of Developmentally Retarded Mutant 1 (DRM1) (Figure 1; Tables S2–S5). In all of these genes, most of the methylated sites occurred at CG sites (Table S3). The CHG and CHH sites were generally not methylated among 25 of the 27 genes, while *DOMAINS REARRANGED METHYLTRANSFERASE 2* (*DRM2*) and *AGAMOUS-LIKE 28* (*AGL28*) showed variable CHG and CHH methylation sites among different *A. thaliana* populations (Tables S4 and S5). However, methylation levels in the coding regions of only six genes—*FVE*, *FY*, *FLD*, *PEP*, *HDA5* and *PRP39-1*—were related to flowering time and total number of leaves after flowering (Figure 1, Table S6) in the different *A. thaliana* accessions (*p* < 0.05). The *A. thaliana* accessions with higher methylation levels of the six genes tended to flower later than those with the lower methylation levels of the genes. The CG methylation levels in the six genes were positively correlated (*p* < 0.05) with flowering time and leaf number (Figure 2, Table S7), whereas the methylation levels of CHG and CHH had no correlations with these characteristics (Tables S8 and S9). The other four downstream genes in the autonomous pathway showed low methylation levels, and the methylation levels of the coding regions of the other 17 genes were not correlated with flowering time or leaf number (Figure 1, Tables S6–S9). The methylation levels of the promoters of the 27 genes were not related to flowering time or leaf number (Tables S10 and S11). The methylation level of the coding regions of the *FWA* gene and its promoter region showed no correlation with flowering time (Figure S2). DNA polymorphisms of *FVE*, *FY*, *FLD*, *PEP*, *HDA5* and *PRP39-1* showed no significant influence on their methylation levels (Figure S3). The results suggested that CG coding region methylation of the *FVE*, *FY*, *FLD*, *PEP*, *HDA5* and *PRP39-1* genes was likely to be involved in the regulation of flowering time in *A. thaliana*.

### 2.2. Relationship between the Methylation Level and Expression Level of Autonomous Pathway Genes

**Figure 3:**
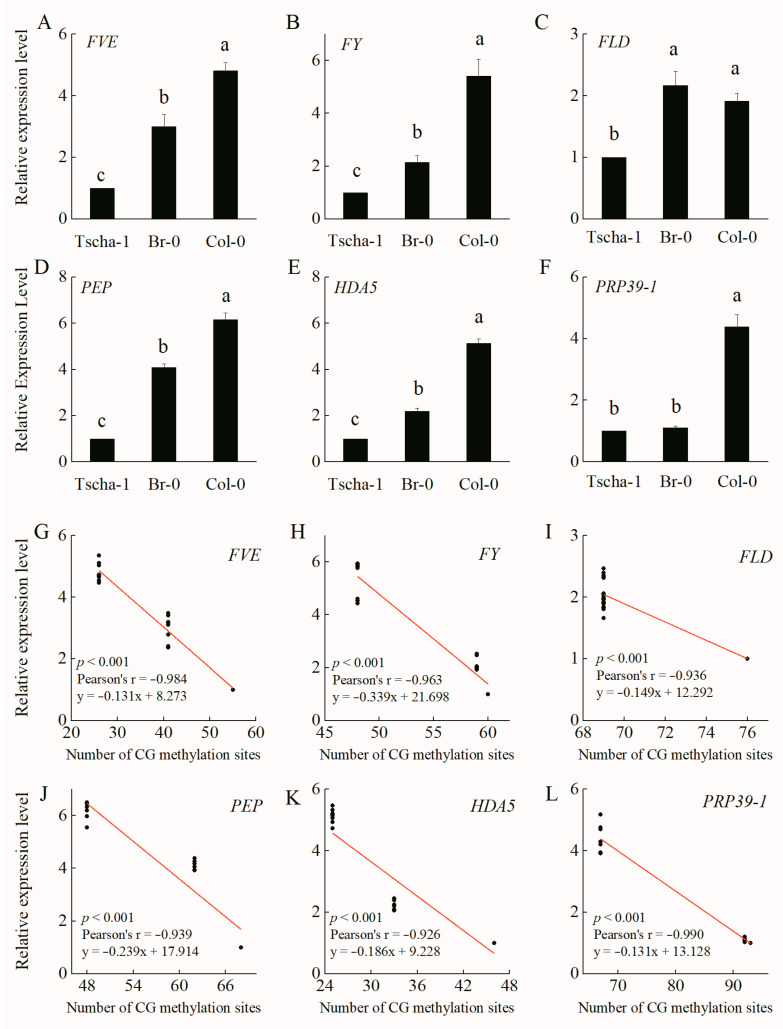
Relative expression levels of the *FVE* (**A**), *FY* (**B**), *FLD* (**C**), *PEP* (**D**), *HDA5* (**E**) and *PRP39-1* (**F**) genes and correlations with coding region methylation levels among Col-0, Br-0 and Tscha-1 (**G**–**L**). The different letters indicate significant differences among the different accessions, *p* < 0.05.

Compared with the middle-flowering accession Br-0 and the late-flowering accession Tscha-1, the early-flowering *A. thaliana* accession Col-0 presented the highest expression levels of *FVE*, *FY*, *FLD*, *PEP*, *HDA5* and *PRP39-1*, while Tscha-1 had the lowest expression levels (Figure 3). There were no significant difference in the expression level of *FLD* between Col-0 and Br-0 (Figure 3C) and in the expression level of *PRP39-1* between Br-0 and Tscha-1 (Figure 3F). The results were consistent with the methylation levels of the two genes among these accessions (Table S2). The expression levels of the six genes were negatively correlated (*p* < 0.001) with the CG methylation levels in Col-0, Br-0 and Tscha-1 (Figure 3G–L). As CHG and CHH were generally not methylated in *FVE*, *FY*, *FLD*, *PEP*, *HDA5* and *PRP39-1*, we did not analyze the correlations between the expression levels and the CHG and CHH methylation levels (Tables S4 and S5). Because only a portion of the 27 *A. thaliana* accessions were used for transcriptome determination in the previous study [20,30], a correlation analysis of the autonomous pathway genes of 15 accessions was conducted using transcriptome and epigenome data. The methylation levels of the coding regions of the six genes were correlated with the expression levels (Table S12). RT-qPCR (real-time quantitative polymerase chain reaction) analysis of the four downstream genes (*FLC*, *SOC1*, *LFY* and *AP1*) showed that among the accessions of Col-0, Br-0 and Tscha-1, late-flowering Tscha-1 showed the highest expression of *FLC*, which negatively regulates *A. thaliana* flowering (Figure S4A). Early-flowering Col-0 had the highest expression levels of the *SOC1*, *LFY* and *AP1* genes, while Tscha-1 had the lowest expression levels (Figure S4B–D). 

### 2.3. Reduction in the Flowering Variation Caused by 5-azaC Treatment

After the 5-azaC treatment, the diversity in flowering time across the different *A. thaliana* accessions decreased (Figure S5A), ranging from 22 to 47.6 days. This treatment led to significant hypomethylation in the coding regions of *FVE*, *FY*, *FLD*, *PEP*, *HDA5*, and *PRP39-1* genes compared to untreated controls (Table S13). Methylation levels in these genes decreased by 14–56% in the 5-azaC-treated Col-0, Br-0, and Tscha-1 accessions, compared to their controls. Notably, this decrease in methylation was primarily attributed to a reduction in the number of methylated CG sites (Table S13). Furthermore, a reduction in the variability of DNA methylation levels was observed across the three accessions for each of the six genes examined (Figure S5B). This suggests that 5-azaC treatment may lead to a more uniform methylation pattern among the accessions. The relative expression levels of these genes (*FVE*, *FY*, *FLD*, *PEP*, *HDA5*, and *PRP39-1*) increased among Col-0, Br-0 and Tscha-1 after 5-azaC treatment; however, the range of variation in these relative expression levels decreased (Figure S5C). The relative expression levels of these six genes were correlated with both the total number of methylated sites and the number of CG methylated sites in the gene coding regions after 5-azaC treatment (*p* < 0.05) (Figure S5D). These results indicated that the 5-azaC treatment reduced the flowering diversity, probably due to decreases in both the variation in the methylation level and the relative expression levels of the *FVE*, *FY*, *FLD*, *PEP*, *HDA5* and *PRP39-1* genes.

### 2.4. Restoration of the Flowering Phenotype of fve-3 by Expression of FVE or FVE(CS)

**Figure 4:**
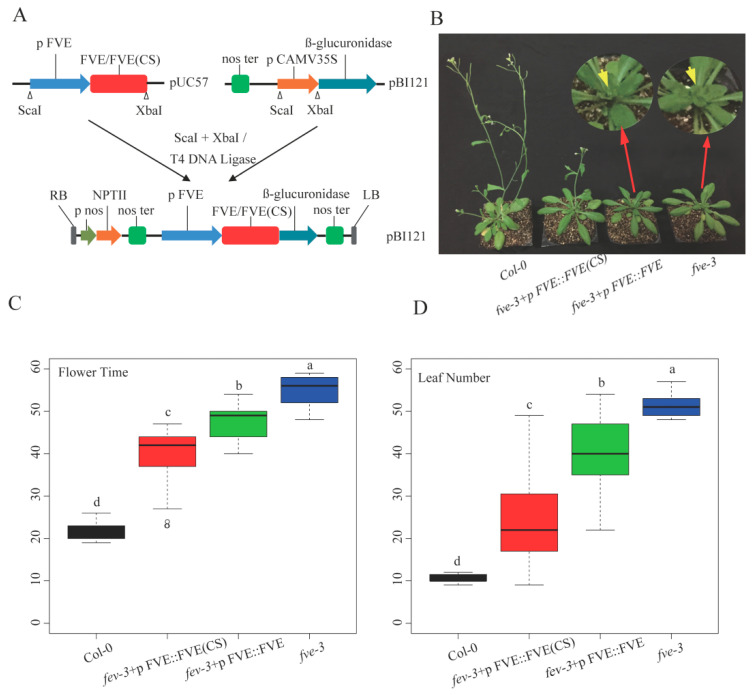
Expression analysis of *FVE*(*CS*) and *FVE* in *fve-3* and their flowering phenotype. (**A**), Construction of *FVE* and *FVE*(*CS*) plant expression vectors; (**B**), Phenotypes of six-week-old seedlings of Col-0, *fve-3*+p*FVE*::*FVE*(*CS*), *fve-3*+p*FVE*::*FVE* and *fve-3*; (**C**), Flowering time differences among these plants; and (**D**), Leaf number differences among these plants. The different letters indicate significant differences among the different plants, *p* < 0.05.

*FVE* and the modified *FVE* gene sequence named *FVE*(*CS*) (reduced theoretical methylation sites in the gene coding region, details in Section 4.4) shared similar CAI values of 0.69 and 0.66, respectively. Five individual *A. thaliana* plants expressing *FVE* (named *fve-3*+p*FVE*::*FVE*) and nine individual plants expressing *FVE*(*CS*) (named *fve-3*+p*FVE*::*FVE*(*CS*)) were obtained (Figure 4A). The two types of transgenic plants (*fve-3*+p*FVE*::*FVE* and *fve-3*+p*FVE*::*FVE*(*CS*)) flowered significantly earlier (at 23 days and 40 days after planting, respectively) than the *fve-3* mutants (48 days after planting). Flowering occurred much earlier in *fve-3*+p*FVE*::*FVE*(*CS*) than in *fve-3*+p*FVE*::*FVE*. However, the transgenic plants still flowered later than the Col-0 plants (21.6 days) (Figure 4B,C). At the flowering stage, the *fve-3*+p*FVE*::*FVE*(*CS*) and *fve-3*+p*FVE*::*FVE* plants had fewer leaves than the *fve-3* mutants, but more leaves than the Col-0 plants (Figure 4D). The results indicated that *FVE*(*CS*) partially restored the function of *FVE* in *fve-3* relative to Col-0. 

### 2.5. Expression Levels of the Autonomous Pathway Genes in fve-3+pFVE::FVE(CS) and fve-3+pFVE::FVE Plants

**Figure 5:**
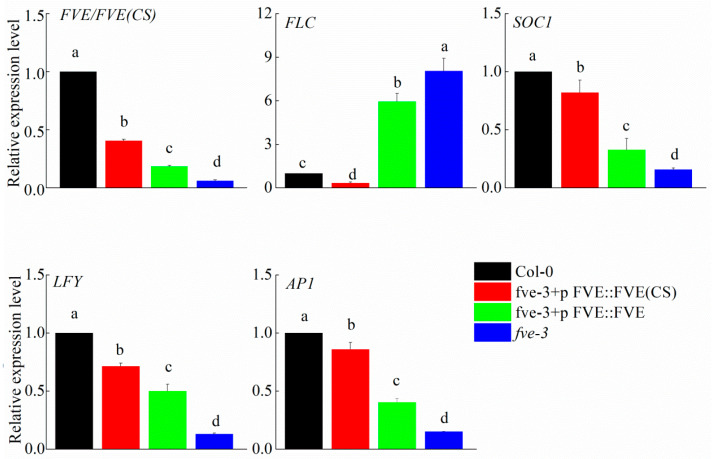
RT-qPCR analysis of the autonomous pathway genes *FVE*, *FLC*, *SOC1*, *LFY* and *AP1* of Col-0, *fve-3*+p*FVE*::*FVE*(*CS*), *fve-3*+p*FVE*::*FVE* and *fve-3*. The letters indicate significant differences among the different transgenic plants, *p* < 0.05.

The relative expression level of *FVE* in Col-0 was significantly higher than in the transgenic plants, and the expression of *FVE* was nearly undetectable in *fve-3* (Figure 5). The relative expression level of *FVE*(*CS*) in *fve-3*+p*FVE*::*FVE*(*CS*) was significantly higher than the expression level of *FVE* in *fve-3*+p*FVE*::*FVE* (Figure 5). Expression of the downstream gene *FLC* was nearly undetectable in the Col-0 and *fve-3*+p*FVE*::*FVE*(*CS*) plants, and *FLC* was expressed at a low level in the *fve-3*+p*FVE*::*FVE* individuals, whereas the *fve-3* mutants showed the highest expression level (Figure 5). The positive flowering regulators *SOC1*, *LFY* and *AP1* were barely expressed in the *fve-3* mutants; the *fve-3*+p*FVE*::*FVE*(*CS*) plants had higher expression levels of these genes than in the *fve-3*+p*FVE*::*FVE* plants, while the Col-0 plants presented the highest expression level of these genes (Figure 5). Differences in the expression of the *FVE*, *FLC*, *SOC1*, *LFY* and *AP1* genes may lead to differences in flowering time between these plants.

### 2.6. Methylation Levels of FVE and FVE(CS) in Col-0 and Transgenic Plants

**Figure 6:**
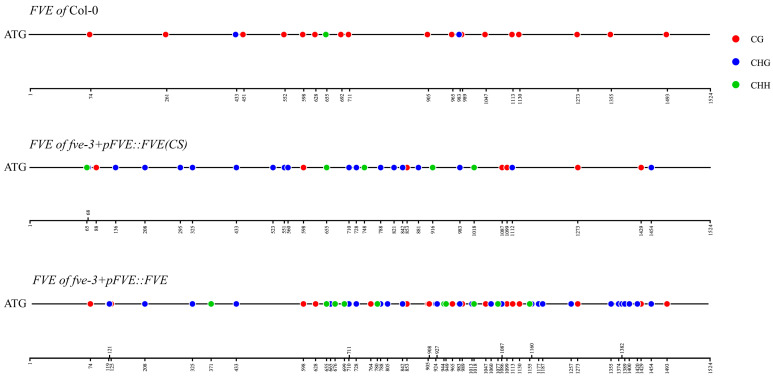
Methylated sites of *FVE* and *FVE*(*CS*) genes in Col-0, *fve-3*+p*FVE*::*FVE* and *fve-3*+p*FVE*::*FVE*(*CS*) plants.

The *FVE*(*CS*) and *FVE* genes had methylated sites of 30 and 57, respectively, in the transgenic plants (Figure 6; Table S14). There were only 20 methylated sites within the *FVE* gene in the Col-0 plants, which is fewer than the corresponding number in the transgenic plants (Figure 6; Table S14). Only five methylated sites within the *FVE*(*CS*) gene in the *fve-3*+p*FVE*::*FVE*(*CS*) plants were the same as those in the Col-0 plants, and the other 25 methylated sites were novel and not methylated in Col-0 (Figure 6; Table S14). However, 16 methylated sites within the *FVE* gene in the *fve-3*+p*FVE*::*FVE* plants were the same as those in the Col-0 plants. The other 39 methylated sites within the *FVE* gene were novel compared to those in Col-0 (Figure 6; Table S14). The *FVE*(*CS*) gene in the *fve-3*+p*FVE*::*FVE*(*CS*) plants had more CHG and CHH methylated sites and fewer CG methylated sites than the *FVE* gene in Col-0 (Figure 6; Table S14). The *FVE* gene in the *fve-3*+p*FVE*::*FVE* plants had higher methylation levels of CG, CHG and CHH than the *FVE* gene in Col-0 (Figure 6; Table S14). The higher methylation levels of the *FVE* and *FVE*(*CS*) genes were associated with the lower expression levels and late flowering of the *fve-3*+p*FVE*::*FVE* and *fve-3*+p*FVE*::*FVE*(*CS*) compared to Col-0 plants.

## 3. Discussion

In this study, all 27 *A. thaliana* accessions were grown in a controlled environment that eliminated the influence of environmental factors [9]. However, different *A. thaliana* accessions still showed various flowering times (Table S1), indicating that the flowering of *A. thaliana* was also affected by endogenous factors [34]. DNA methylation has been shown to be involved in the regulation of flowering time in *A. thaliana*, such as *FWA* [18,19]. However, the methylation level of the *FWA* gene showed no correlation with the flowering time of the 27 *A. thaliana* accessions (Figure S2). The CG methylation levels of six genes (*FVE*, *FY*, *FLD*, *PEP*, *HDA5* and *PRP39-1*) in the autonomous pathway were significantly correlated with the corresponding gene expressions and the phenotypic differences in flowering time, indicating that CG coding region methylation of the six genes might be associated with the regulation of the expressions, leading to variations in flowering time. In addition, DNA polymorphisms of these six genes showed no significant influence on their methylation levels (Figure S3). Our results suggested that DNA methylation of certain key genes might play important roles in flowering time variation in *A. thaliana*.

These six genes act as upstream components in the autonomous pathway, indicating that the pathway’s function is likely influenced by the DNA methylation of these regulatory genes. For all 27 upstream genes, most methylated sites were CG sites, consistent with the notion that CG gene-body methylation is a common feature of animal and plant genomes [35,36]. Studies have shown that *FY*, *PEP*, *PRP39-1*, *FVE*, *FLD*, *FLC* and *HDA5* are involved in RNA processing, transcription, post-transcription and chromatin modification [5]. We hypothesized that the methylation levels of the upstream regulators of the six genes studied here (*FVE*, *FY*, *FLD*, *PEP*, *HDA5* and *PRP39-1*) could be closely related to flowering time variation and their expression levels.

All the downstream genes in the autonomous pathway generally had lower methylation levels and were not related to flowering time (Figure 1; Table S2). Considering the methylation levels of the *ICE1* gene in crofton weed, *A. thaliana* and weedy rice [24,26,27], we infer that the DNA methylation of upstream regulators likely plays a role in the pathways responsible for environmental responses. This is consistent with the known function of DNA methylation, in which plants respond quickly to different environmental conditions through DNA methylation independent of gene sequence changes, leading to differential expressions [37,38]. This process greatly benefits plant survival in different environments, as changes in DNA methylation are much easier and more flexible than gene mutations [39,40]. Responding to environmental changes through the methylation of upstream regulators in transduction pathways could improve the sensitivity of plant responses and benefit plant survival.

In this study, the complemented lines *fve-3*+p*FVE*::*FVE*(*CS*) and *fve-3*+p*FVE*::*FVE* showed distinct patterns in terms of DNA methylation, flowering time and gene expression. The different methylation levels of *FVE*(*CS*) and *FVE* corresponded to the differential expressions of the downstream genes in the autonomous pathway and flowering time variation. Since *FVE* and *FVE*(*CS*) shared similar CAI values, the difference in codon usage caused by degenerate codon substitution cannot explain the remarkable difference in translation efficiency. These results suggested that methylation of the *FVE* gene may be involved in flowering time variation in *A. thaliana*. The flowering phenotypes of Col and *fve-3* were both highly synchronized; in contrast, the spreading of flowering time over a broader time interval in the transgenic plants could be a hallmark of an epigenetic effect. Both the *FVE*(*CS*) and *FVE* genes in the transgenic plants *fve-3*+p*FVE*::*FVE*(*CS*) and *fve-3*+p*FVE*::*FVE* were methylated, and the methylation levels of the *FVE*(*CS*) and *FVE* gene were higher in the transgenic plants than in the Col-0 plants. The results implied that expressing *FVE*(*CS*) or *FVE* in *fve-3* could only partially restore the flowering phenotype. Transgenes tend to be hypermethylated in transgenic plants; this hypermethylation could be a protective mechanism in plants. However, modification of the gene sequence through codon degeneracy substitution to reduce the number of cytosine nucleotides can relatively avoid hypermethylation of transgenes, although methylation of plants expressing *FVE*(*CS*) appears more stochastic compared with those expressing *FVE* (Figure 4C). Thus, it may be possible to partially regulate the flowering of plants via this technology. In addition, crop production and environmental adaptability may be improved by changing the relevant gene methylations using this relatively safe and effective approach—codon degeneracy substitution—in the future.

In conclusion, our study indicates that methylation of the *FVE* gene is likely to be associated with flowering time variations in *A. thaliana* through regulating the gene expression. The AuPC promotes plant flowering by repressing the *FLC* expression, and each of the AuPC components could also be involved in the plant flowering regulation independently [14]. The AuPC-based molecular mechanism provides a precise network for plant flowering regulation. Methylation alterations of the key genes seem to be an effective way to adapt to a changing environment rapidly. The question of whether key gene methylation is involved in other plant phenotypic variations needs to be further studied.

## 4. Materials and Methods

### 4.1. Plant Materials, Growth Conditions and Flowering Time Statistical Analysis

In this study, 27 *A. thaliana* accessions were selected to represent a wide range of flowering times, from early-flowering to late-flowering phenotypes. These accessions, which exhibited flowering times ranging from 19 to 55.4 days under our experimental conditions, were purchased from the Arabidopsis Biological Resource Center (Table S1). The plants were grown in a controlled-environment chamber (23 °C, 16/8-h photoperiod, 120 μmol photons m^−2^ s^−1^, humidity 60–70%). The descendants of a single mother plant of each *A. thaliana* accession were used in this study. The seeds were sterilized and cold-stratified in the dark at 4 °C for 2 days before sowing, followed by transplant on Murashige and Skoog (MS) media consisting of 0 (WT) or 50 µM 5-azaC (T-5azaC). After 7 days, the seedlings were transferred to plastic pots filled with a 3.5:1 mixture of peat moss–vermiculite and grown under the conditions described above. The flowering times of 15 individual plants and the total number of leaves were observed for each accession, and the experiment was repeated three times. Bivariate correlation analysis of flowering time, leaf number and DNA methylation level was conducted using SPSS 20 software (IBM, Armonk, NY, USA). R 3.6.3(R Foundation for Statistical Computing, Vienna, Austria) and Origin 8 (OriginLab Corporation, Northampton, MA, USA) were used for statistical analysis, and the Mann–Whitney U test was used to analyze significant differences at the level of *p* < 0.05 in flowering time between the WT and T-5azaC accessions. Linear correlation analysis of the relative expression level and number of methylated sites was performed using Origin 8 (*p* < 0.05). The correlations between DNA methylation and relative expression levels were analyzed using SPSS 20 software, and the results were plotted with the Origin 8 and R 3.6.3 (*p* < 0.05).

### 4.2. Extraction of Epigenome and Transcriptome Data and Analysis of the DNA Methylation of Autonomous Pathway Genes and Its Correlations with Flowering Time and Leaf Numbers

Epigenomic data of different *A. thaliana* accessions collected by Schmitz et al. [41] were downloaded. In their study, custom algorithms were used to identify mC sites as described by Lister et al. [42]. The methylation information for the coding regions and their promoter regions (1500 bp upstream of ATG) of autonomous pathway genes from 27 *A. thaliana* accessions was extracted using the R 3.6.3. In addition, the CG, CHG and CHH (where H is A, C, or T) methylated sites in the coding regions of the genes were determined. The RNA-Seq data of GSE43858 [41] and GSE80744 [20] were downloaded from The Gene Expression Omnibus (GEO). The autonomous pathway gene expression level was estimated using FPKM (fragments per kilobase per million reads) values, which were extracted using R 3.6.3. The correlations among DNA methylation, flowering time, the total number of leaves after flowering and gene expression levels of the different *A. thaliana* accessions were determined using SPSS 20 software, and the data were plotted via R 3.6.3. GWAS (Genome Wide Association Study) was conducted using 1001 Genomes Project public tools: GWAPP (https://gwas.gmi.oeaw.ac.at/, accessed on 18 November 2022) with the “1001 Fullsequence Dataset”. The gene body methylation levels of *FVE*, *FY*, *FLD*, *PEP*, *HDA5* and *PRP39-1* were retrieved from http://neomorph.salk.edu/downloads/1001/ (accessed on 22 June 2021). A total of 23 *A. thaliana* ecotypes were included in the GWAS analysis, as methylome or genome data were missing for Tu-0, Hey-1, Ty-0 and Rd-0.

### 4.3. Expression Dectection of Autonomous Pathway Genes

In this study, three *A. thaliana* accessions were used for Real-time quantitative polymerase chain reaction (RT-qPCR) analysis: the early-flowering accession Col-0, middle-flowering accession Br-0 and late-flowering accession Tscha-1. The expression levels of six upstream methylated genes, *FLOWERING LOCUS VE* (*FVE*), *FLOWERING LOCUS Y* (*FY*), *FLOWERING LOCUS D* (*FLD*), *PEPPER* (*PEP*), *HISTONE DEACETYLASE5* (*HDA5*) and *Pre-mRNA Processing Protein39-1* (*PRP39-1*) correlated with flowering time, and those of four downstream genes in the autonomous pathway, *FLC*, *SUPPRESSOR OF OVEREXPRESSION OF CONSTANS 1*/*AGAMOUS-LIKE20* (*SOC1*/*AGL20*), *LEAFY* (*LFY*), and *APETALA1* (*AP1*), were determined by RT-qPCR with the primers listed in Table S15. The expression levels of the six methylated genes were also determined in 5-azaC-treated Col-0, Br-0 and Tscha-1 plants. Three-week-old Col-0, Br-0 and Tscha-1 seedlings were used for RT-qPCR analysis. The expression levels were normalized to that of the *beta*-*actin* gene and calculated for each population using the 2^−ΔΔCt^ method [43]. Fold changes in the expression of autonomous pathway genes in the 5-azaC-treated plants were calculated relative to their expressions in untreated plants. Three biological replicates were included for each gene, and three different seedlings were used for RT-qPCR in each replicate. The differences were analyzed by one-way analysis of variance (ANOVA) using SPSS 20 software.

### 4.4. Codon Degeneracy-Based Nucleotide Substitution of the FVE Gene and Transgenic Verification

According to the codon degeneracy of amino acids, the nucleotides CG, CHG and CHH of the *FVE* gene were substituted to minimize its methylation sites. There were 225 cytosine nucleotides within the *FVE* gene that could theoretically be methylated, and a total of 127 cytosine nucleotides were substituted according to codon degeneracy (Figure S6). The modified *FVE* gene sequence was named as *FVE*(*CS*) and was synthesized by Sangon Biotech (201611, Shanghai, China). The codon adaptation index (CAI) value was calculated using EMBOSS (http://www.bioinformatics.nl/emboss-explorer/, accessed on 13 May 2019). *FVE* and *FVE*(*CS*) were used for plant expression vector construction and *A. thaliana fve-3* [5] transformation.

The primers used for p*FVE*-*FVE*-pBI121 and p*FVE*-*FVE*(*CS*)-pBI121 vector construction are listed in Table S16. The vectors were then used for *A. thaliana* transformation via the floral dip method [44]. After transgenic *A. thaliana* seeds were harvested, the seeds were cultivated on MS media consisting of 50 μg ml^−1^ kanamycin without vernalization for screening. Transgenic plants with a single copy of the exogenous gene were identified and used for subsequent experiments. The flowering times and leaf numbers of 15 individuals each of Col-0, *fve-3* plants and homozygotes of the third generation of each transgenic individual were determined. Three-week-old young leaves of the earliest flowering plants of Col-0, *fve-3*+p*FVE*::*FVE*(*CS*), *fve-3*+p*FVE*::*FVE* and *fve-3* were selected, and three individuals of each plant were used for RT-qPCR analysis and methylation determination.

### 4.5. Methylation Determination

Ten individuals of Col-0, *fve-3* and the two transgenic plants were used for methylation determination. The methylated sites in the *FVE* and *FVE*(*CS*) genes were determined using bisulfite modification (EZ DNA Methylation-GoldTM Kit) with the primers listed in Table S17. The PCR products were ligated into pMD^TM^19-T vectors (pMDTM^19^-T Vector Cloning Kit, TaKaRa, Tokyo, Japan), and 10 clones were sequenced for each sample. The obtained sequences (GenScript USA Inc., Nanjing, China) of each sample were compared with the original *FVE* sequence or *FVE*(*CS*) sequence, and a converted cytosine indicated an unmethylated site. The thresholds for labeling a cytosine as methylated in the CG, CHG or CHH contexts were set to 50%, 20% and 20%, respectively. Six individuals each from the 5-azaC-treated Col-0, Br-0 and Tscha-1 accessions were also used for the *FVE*, *FY*, *FLD*, *PEP*, *HDA5* and *PRP39-1* methylation determination using the primers listed in Table S17.

## Data Availability

The original contributions presented in the study are included in the article/Supplementary Material, further inquiries can be directed to the corresponding author.

## Supplementary Materials

The following supporting information can be downloaded at: https://www.mdpi.com/article/10.3390/ijms25137478/s1.
