# Supplementary material for: DNA Methylation of the Autonomous Pathway Is Associated with Flowering Time Variations in Arabidopsis thaliana
Source: Int J Mol Sci. 2024 Jul 8;25(13):7478. doi: 10.3390/ijms25137478 (PMC11242178; doi:10.3390/ijms25137478)
Supplement: Supplementary file 1 [file ijms-25-07478-s001.zip › ijms-3081151-supplementary.pdf]

Supplementary

Table S1. Information on the *A. thaliana* accessions, flowering time and total number of leaves after flowering.

TAIR accessions	Abbreviated name	Flowering time (d)	Leaf number
CS28652	Pro-0	19.0±0.0	9.7±0.9
CS76537	Kz-9	21.0±1.0	10.6±0.8
CS28573	Nw-0	21.0±1.0	10.4±0.5
CS6845	Rd-0	21.0±1.0	10.7±0.8
CS6885	Wa-1	24.0±0.0	12.1±0.3
CS6905	Hs-0	24.0±1.0	12.4±0.6
CS6105	Kelsterbach-4	24.0±0.0	11.9±0.7
CS6673	Col-0	27.0±2.0	13.7±1.0
CS6675	Cvi-0	27.0±0.0	13.7±0.9
CS28128	Ca-0	28.0±2.0	14.3±0.7
CS28096	Bs-1	29.0±0.0	14.8±0.7
CS6797	Ms-0	29.4±1.8	14.3±1.1
CS6630	Bsch-0	30.0±0.9	14.7±1.1
CS6923	Ema-1	30.0±1.5	15.3±0.9
CS6732	Gy-0	31.0±0.8	15.7±0.6
CS6865	Stw-0	33.8±2.0	16.7±1.0
CS6600	Aa-0	35.0±1.0	17.7±0.8
CS6723	Gr-1	38.0±0.0	18.5±0.7
CS6862	Sp-0	39.0±0.8	19.7±0.7
CS6875	Tu-0	42.0±2.3	20.9±1.1
CS6626	Br-0	45.0±1.0	22.6±1.0
CS28344	Hey-1	45.0±0.8	22.5±1.0
CS28597	Per-1	45.0±2.0	22.5±1.3
CS28786	Ty-0	45.4±1.8	22.6±1.1
CS6867	Ta-0	46.5±1.1	23.9±1.0
CS22518	Tscha-1	54.0±2.3	26.7±0.9
CS6852	Se-0	55.4±1.2	27.3±0.9

TAIR, The Arabidopsis Information Resource. Data are shown as mean±standard errors.

Table S2. Number of methylated sites of autonomous pathway gene coding regions of different *A. thaliana* accessions.

Population	Number of methylated sites													
	FVE	***FY***	***FLD***	***PEP***	***HDA5***	***PRP39-1***	***FCA***	***FLK***	***FPA***	***LD***	***PCFS4***	***DRM2***	***HDA6***	***PRMT5***
	AT2G19520	AT5G13480	AT3G10390	AT4G26000	AT5G61060	AT1G04080	AT4G16280	AT3G04610	AT2G43410	AT4G02560	AT4G04885	AT5G14620	AT5G63110	AT4G31120
Aa-0	29	49	56	31	48	88	79	35	43	45	70	50	8	59
Br-0	41	59	69	33	62	92	107	54	81	82	81	67	18	50
Bs-1	23	43	46	15	54	76	54	41	43	61	52	37	14	17
Bsch-0	37	52	60	25	30	77	84	45	66	80	71	50	6	70
Ca-0	33	58	42	19	49	86	83	43	71	57	64	38	10	53
Col-0	20	49	69	25	48	67	78	45	51	66	67	33	1	51
Cvi-0	19	31	33	9	18	40	47	19	36	51	33	56	0	13
Ema-1	36	66	59	34	41	76	78	57	62	74	85	29	10	69
Gr-1	13	44	46	16	51	70	73	18	41	81	46	32	15	39
Gy-0	25	52	54	35	42	85	78	35	56	55	67	30	7	54
Hey-1	22	62	64	36	44	74	83	44	66	52	64	58	0	56
Hs-0	28	44	56	32	40	83	88	37	66	81	79	61	14	54
Kelsterbach-4	23	42	51	21	32	79	64	48	74	61	63	62	7	36
Kz-9	22	40	50	16	25	62	91	39	47	63	55	86	7	62
Ms-0	46	27	69	34	53	61	93	56	74	66	78	48	23	46
Nw-0	37	59	67	21	61	100	85	54	78	81	93	77	12	45
Per-1	25	47	49	38	44	69	73	37	41	108	69	30	5	58
Pro-0	20	49	37	20	34	47	80	37	62	71	60	31	10	21
Rd-0	19	32	18	19	31	59	45	25	28	42	40	12	7	25
Se-0	31	53	58	45	44	82	84	53	60	73	84	64	20	49
Sp-0	32	47	57	36	38	84	2	40	79	5	2	25	8	65
Stw-0	25	50	39	26	38	79	88	37	38	67	48	37	10	57
Ta-0	42	67	73	44	54	79	106	56	78	92	85	72	14	58
Tscha-1	59	60	78	46	70	93	118	44	84	81	84	77	21	63
Tu-0	34	30	62	34	47	87	93	47	67	95	72	80	8	59
Ty-0	33	71	73	46	55	101	102	64	92	71	92	62	17	52
Wa-1	22	52	60	26	40	80	85	46	53	67	63	63	15	65

Table S2 (continued). Number of methylated sites of autonomous pathway gene coding regions of different *A. thaliana* accessions.

Population	Number of methylated sites												
	PRMT10	***REF6***	***AGL28***	***CK2***	***PP2A-B'γ***	***SYP22***	***ESD4***	***DBP1***	***DRM1***	***FLC***	***SOC1***	***LFY***	***AP1***
	AT1G04870	AT3G48430	AT1G01530	AT2G23080	AT4G15415	AT5G46860	AT4G15880	AT2G25620	AT5G15380	AT5G10140	AT2G45660	AT5G61850	AT1G69120
Aa-0	6	113	48	24	52	18	18	12	4	1	0	0	0
Br-0	14	116	40	30	67	18	15	22	0	2	0	0	0
Bs-1	23	109	41	39	44	0	18	13	0	5	0	2	0
Bsch-0	20	80	60	36	47	13	14	12	0	0	0	0	0
Ca-0	20	92	43	29	42	13	3	15	8	0	0	0	0
Col-0	8	74	39	23	37	1	8	18	0	0	0	0	0
Cvi-0	0	51	46	20	14	0	0	15	0	0	0	0	0
Ema-1	23	114	47	26	40	19	14	17	1	0	1	0	0
Gr-1	4	64	45	13	34	16	14	5	2	1	0	0	1
Gy-0	15	73	42	19	49	13	20	14	4	0	0	0	0
Hey-1	15	95	39	32	47	0	29	16	2	0	0	0	0
Hs-0	2	94	53	31	49	9	3	21	1	0	0	0	2
Kelsterbach-4	17	83	45	28	62	1	9	11	0	0	0	0	0
Kz-9	14	91	35	16	39	5	10	11	0	0	1	0	0
Ms-0	6	126	36	30	53	15	10	16	13	0	0	0	0
Nw-0	21	122	39	28	81	2	18	28	4	0	0	3	0
Per-1	6	109	28	10	41	9	4	13	4	0	0	0	0
Pro-0	0	68	36	21	34	13	20	8	2	2	0	0	0
Rd-0	8	45	18	14	32	2	5	15	0	0	0	0	0
Se-0	17	107	12	27	70	11	7	11	0	1	0	0	0
Sp-0	9	96	37	19	14	5	0	18	4	13	0	0	0
Stw-0	15	79	49	24	48	12	12	5	0	7	1	0	0
Ta-0	12	105	27	30	79	17	20	22	6	9	0	0	0
Tscha-1	6	84	37	24	66	2	14	14	0	0	0	0	0
Tu-0	6	97	43	27	75	2	17	15	10	1	0	0	0
Ty-0	20	120	32	24	56	27	24	21	1	0	0	0	0
Wa-1	11	90	52	27	55	10	17	12	0	0	0	0	0

Table S3. Number of methylated CG sites in the coding regions of autonomous pathway genes of different *A. thaliana* accessions.

Population	Number of CG methylated sites													
	FVE	***FY***	***FLD***	***PEP***	***HDA5***	***PRP39-1***	***FCA***	***FLK***	***FPA***	***LD***	***PCFS4***	***DRM2***	***HDA6***	***PRMT5***
	AT2G19520	AT5G13480	AT3G10390	AT4G26000	AT5G61060	AT1G04080	AT4G16280	AT3G04610	AT2G43410	AT4G02560	AT4G04885	AT5G14620	AT5G63110	AT4G311 20
Aa-0	29	49	56	31	48	88	79	35	43	45	70	22	8	59
Br-0	41	59	69	33	62	92	105	54	81	80	81	45	18	50
Bs-1	23	43	46	15	54	76	53	41	43	61	51	25	14	17
Bsch-0	37	51	58	25	30	77	82	44	66	80	71	30	5	70
Ca-0	33	58	42	19	49	86	83	43	71	57	63	25	10	53
Col-0	26	48	69	25	48	67	78	45	51	66	66	27	1	51
Cvi-0	19	31	33	9	18	40	44	19	36	51	33	22	0	13
Ema-1	36	66	59	34	41	76	78	57	61	74	85	29	10	69
Gr-1	13	44	46	16	50	70	73	18	41	79	46	16	15	39
Gy-0	25	52	54	35	42	85	78	35	56	55	67	21	7	54
Hey-1	22	62	64	36	44	74	83	44	66	52	64	40	0	56
Hs-0	28	44	55	32	40	83	88	37	66	81	79	39	14	53
Kelsterbach -4	23	42	51	21	32	75	63	48	74	61	63	30	7	36
Kz-9	22	39	49	16	25	62	90	38	47	63	54	31	7	62
Ms-0	46	27	69	34	53	61	92	56	74	66	77	31	23	46
Nw-0	37	57	65	21	61	99	83	54	77	78	93	33	12	45
Per-1	25	47	49	38	44	69	72	37	41	70	69	26	5	58
Pro-0	20	49	37	20	34	47	77	37	62	71	59	21	10	21
Rd-0	19	32	18	19	31	59	45	25	28	42	40	7	7	25
Se-0	31	53	58	45	44	82	82	53	60	73	84	27	20	49
Sp-0	32	47	57	36	38	83	1	40	79	5	2	24	8	64
Stw-0	25	50	39	26	38	79	85	37	38	66	48	20	10	57
Ta-0	42	67	73	44	54	79	106	56	78	92	85	42	14	58
Tscha-1	55	60	76	46	68	93	114	44	82	81	83	36	21	62
Tu-0	34	29	62	34	46	86	93	46	67	93	72	37	8	59
Ty-0	33	70	73	46	55	100	96	64	91	70	91	29	17	52
Wa-1	22	52	60	26	39	80	85	46	53	67	63	29	15	65

Table S3 (continued). Number of methylated CG sites in the coding regions of autonomous pathway genes of different *A. thaliana* accessions.

Population	Number of CG methylated sites												
	PRMT10	***REF6***	***AGL28***	***CK2***	***PP2A-B'γ***	***SYP22***	***ESD4***	***DBP1***	***DRM1***	***FLC***	***SOC1***	***LFY***	***AP1***
	AT1G04870	AT3G48430	AT1G01530	AT2G23080	AT4G15415	AT5G46860	AT4G15880	AT2G25620	AT5G15380	AT5G10140	AT2G45660	AT5G61850	AT1G69120
Aa-0	6	113	26	24	52	18	18	12	4	1	0	0	0
Br-0	14	116	26	30	67	18	15	22	0	2	0	0	0
Bs-1	23	109	25	39	44	0	16	13	0	5	0	2	0
Bsch-0	20	80	26	36	47	13	14	12	0	0	0	0	0
Ca-0	20	92	24	29	42	13	3	15	8	0	0	0	0
Col-0	8	74	25	23	37	1	8	18	0	0	0	0	0
Cvi-0	0	51	22	20	14	0	0	15	0	0	0	0	0
Ema-1	23	114	26	26	40	19	14	17	0	0	0	0	0
Gr-1	4	64	25	13	34	16	14	5	2	0	0	0	0
Gy-0	15	73	24	19	49	13	20	14	4	0	0	0	0
Hey-1	15	95	23	32	47	0	29	16	2	0	0	0	0
Hs-0	2	94	27	31	49	9	3	20	1	0	0	0	2
Kelsterbach-4	17	83	26	28	62	1	9	11	0	0	0	0	0
Kz-9	14	91	26	16	39	5	10	10	0	0	1	0	0
Ms-0	6	125	26	30	53	15	10	15	13	0	0	0	0
Nw-0	21	122	26	28	81	2	18	26	4	0	0	3	0
Per-1	6	109	23	10	41	9	4	13	4	0	0	0	0
Pro-0	0	68	23	21	33	13	20	8	2	2	0	0	0
Rd-0	8	45	13	14	32	2	5	15	0	0	0	0	0
Se-0	17	107	0	27	70	11	7	11	0	1	0	0	0
Sp-0	9	96	26	19	14	5	0	18	4	4	0	0	0
Stw-0	15	77	26	24	48	12	12	5	0	3	1	0	0
Ta-0	12	105	23	30	79	17	20	22	6	8	0	0	0
Tscha-1	6	84	25	24	66	2	14	14	0	0	0	0	0
Tu-0	6	93	26	26	75	2	17	15	10	0	0	0	0
Ty-0	20	119	26	24	56	27	23	20	0	0	0	0	0
Wa-1	11	90	26	27	55	10	15	12	0	0	0	0	0

Table S4. Number of methylated CHG sites in the coding regions of autonomous pathway genes of different *A. thaliana* accessions.

Population	Number of CHG methylated sites													
	FVE	***FY***	***FLD***	***PEP***	***HDA5***	***PRP39-1***	***FCA***	***FLK***	***FPA***	***LD***	***PCFS4***	***DRM2***	***HDA6***	***PRMT5***
	AT2G19520	AT5G13480	AT3G10390	AT4G26000	AT5G61060	AT1G04080	AT4G16280	AT3G04610	AT2G43410	AT4G02560	AT4G04885	AT5G14620	AT5G63110	AT4G31120
Aa-0	0	0	0	0	0	0	0	0	0	0	0	27	0	0
Br-0	0	0	0	0	0	0	2	0	0	2	0	22	0	0
Bs-1	0	0	0	0	0	0	1	0	0	0	1	12	0	0
Bsch-0	0	1	2	0	0	0	1	1	0	0	0	20	1	0
Ca-0	0	0	0	0	0	0	0	0	0	0	1	13	0	0
Col-0	2	1	0	0	0	0	0	0	0	0	1	6	0	0
Cvi-0	0	0	0	0	0	0	3	0	0	0	0	34	0	0
Ema-1	0	0	0	0	0	0	0	0	1	0	0	0	0	0
Gr-1	0	0	0	0	1	0	0	0	0	2	0	15	0	0
Gy-0	0	0	0	0	0	0	0	0	0	0	0	9	0	0
Hey-1	0	0	0	0	0	0	0	0	0	0	0	18	0	0
Hs-0	0	0	1	0	0	0	0	0	0	0	0	22	0	0
Kelsterbach-4	0	0	0	0	0	4	1	0	0	0	0	31	0	0
Kz-9	0	1	1	0	0	0	1	1	0	0	1	55	0	0
Ms-0	0	0	0	0	0	0	1	0	0	0	1	17	0	0
Nw-0	0	2	2	0	0	1	2	0	1	3	0	44	0	0
Per-1	0	0	0	0	0	0	0	0	0	38	0	4	0	0
Pro-0	0	0	0	0	0	0	3	0	0	0	1	10	0	0
Rd-0	0	0	0	0	0	0	0	0	0	0	0	5	0	0
Se-0	0	0	0	0	0	0	2	0	0	0	0	37	0	0
Sp-0	0	0	0	0	0	1	1	0	0	0	0	1	0	1
Stw-0	0	0	0	0	0	0	3	0	0	1	0	17	0	0
Ta-0	0	0	0	0	0	0	0	0	0	0	0	30	0	0
Tscha-1	1	0	2	0	2	0	4	0	2	0	1	41	0	1
Tu-0	0	1	0	0	1	1	0	1	0	2	0	43	0	0
Ty-0	0	1	0	0	0	1	6	0	1	1	1	33	0	0
Wa-1	0	0	0	0	1	0	0	0	0	0	0	34	0	0

Table S4 (continued). Number of methylated CHG sites in the coding regions of autonomous pathway genes of different *A. thaliana* accessions.

Population	Number of CHG methylated sites												
	PRMT10	***REF6***	***AGL28***	***CK2***	***PP2A-B'γ***	***SYP22***	***ESD4***	***DBP1***	***DRM1***	***FLC***	***SOC1***	***LFY***	***AP1***
	AT1G04870	AT3G48430	AT1G01530	AT2G23080	AT4G15415	AT5G46860	AT4G15880	AT2G25620	AT5G15380	AT5G10140	AT2G45660	AT5G61850	AT1G69120
Aa-0	0	0	11	0	0	0	0	0	0	0	0	0	0
Br-0	0	0	10	0	0	0	0	0	0	0	0	0	0
Bs-1	0	0	9	0	0	0	2	0	0	0	0	0	0
Bsch-0	0	0	12	0	0	0	0	0	0	0	0	0	0
Ca-0	0	0	8	0	0	0	0	0	0	0	0	0	0
Col-0	0	0	8	0	0	0	0	0	0	0	0	0	0
Cvi-0	0	0	13	0	0	0	0	0	0	0	0	0	0
Ema-1	0	0	9	0	0	0	0	0	0	0	0	0	0
Gr-1	0	0	8	0	0	0	0	0	0	0	0	0	0
Gy-0	0	0	7	0	0	0	0	0	0	0	0	0	0
Hey-1	0	0	7	0	0	0	0	0	0	0	0	0	0
Hs-0	0	0	10	0	0	0	0	1	0	0	0	0	0
Kelsterbach-4	0	0	10	0	0	0	0	0	0	0	0	0	0
Kz-9	0	0	9	0	0	0	0	1	0	0	0	0	0
Ms-0	0	1	9	0	0	0	0	0	0	0	0	0	0
Nw-0	0	0	10	0	0	0	0	2	0	0	0	0	0
Per-1	0	0	5	0	0	0	0	0	0	0	0	0	0
Pro-0	0	0	8	0	0	0	0	0	0	0	0	0	0
Rd-0	0	0	5	0	0	0	0	0	0	0	0	0	0
Se-0	0	0	11	0	0	0	0	0	0	0	0	0	0
Sp-0	0	0	10	0	0	0	0	0	0	5	0	0	0
Stw-0	0	2	9	0	0	0	0	0	0	1	0	0	0
Ta-0	0	0	4	0	0	0	0	0	0	0	0	0	0
Tscha-1	0	0	7	0	0	0	0	0	0	0	0	0	0
Tu-0	0	4	9	1	0	0	0	0	0	0	0	0	0
Ty-0	0	1	6	0	0	0	1	1	1	0	0	0	0
Wa-1	0	0	9	0	0	0	2	0	0	0	0	0	0

Table S5. Number of methylated CHH sites in the coding regions of autonomous pathway genes of different *A. thaliana* accessions.

**Population**	**Number of CHH methylated sites**													
	FVE	***FY***	***FLD***	***PEP***	***HDA5***	***PRP39-1***	***FCA***	***FLK***	***FPA***	***LD***	***PCFS4***	***DRM2***	***HDA6***	***PRMT5***
	AT2G19520	AT5G13480	AT3G10390	AT4G26000	AT5G61060	AT1G04080	AT4G16280	AT3G04610	AT2G43410	AT4G02560	AT4G04885	AT5G14620	AT5G63110	AT4G31120
Aa-0	0	0	0	0	0	0	0	0	0	0	0	1	0	0
Br-0	0	0	0	0	0	0	0	0	0	0	0	0	0	0
Bs-1	0	0	0	0	0	0	0	0	0	0	0	0	0	0
Bsch-0	0	0	0	0	0	0	1	0	0	0	0	0	0	0
Ca-0	0	0	0	0	0	0	0	0	0	0	0	0	0	0
Col-0	1	0	0	0	0	0	0	0	0	0	0	0	0	0
Cvi-0	0	0	0	0	0	0	0	0	0	0	0	0	0	0
Ema-1	0	0	0	0	0	0	0	0	0	0	0	0	0	0
Gr-1	0	0	0	0	0	0	0	0	0	0	0	1	0	0
Gy-0	0	0	0	0	0	0	0	0	0	0	0	0	0	0
Hey-1	0	0	0	0	0	0	0	0	0	0	0	0	0	0
Hs-0	0	0	0	0	0	0	0	0	0	0	0	0	0	1
Kelsterbach-4	0	0	0	0	0	0	0	0	0	0	0	1	0	0
Kz-9	0	0	0	0	0	0	0	0	0	0	0	0	0	0
Ms-0	0	0	0	0	0	0	0	0	0	0	0	0	0	0
Nw-0	0	0	0	0	0	0	0	0	0	0	0	0	0	0
Per-1	0	0	0	0	0	0	1	0	0	0	0	0	0	0
Pro-0	0	0	0	0	0	0	0	0	0	0	0	0	0	0
Rd-0	0	0	0	0	0	0	0	0	0	0	0	0	0	0
Se-0	0	0	0	0	0	0	0	0	0	0	0	0	0	0
Sp-0	0	0	0	0	0	0	0	0	0	0	0	0	0	0
Stw-0	0	0	0	0	0	0	0	0	0	0	0	0	0	0
Ta-0	0	0	0	0	0	0	0	0	0	0	0	0	0	0
Tscha-1	0	0	0	0	0	0	0	0	0	0	0	0	0	0
Tu-0	0	0	0	0	0	0	0	0	0	0	0	0	0	0
Ty-0	0	0	0	0	0	0	0	0	0	0	0	0	0	0
Wa-1	0	0	0	0	0	0	0	0	0	0	0	0	0	0

Table S5 (continued). Number of methylated CHH sites in the coding regions of autonomous pathway genes of different *A. thaliana* accessions.

Population	**Number of CHH methylated sites**												
	PRMT10	***REF6***	***AGL28***	***CK2***	***PP2A-B'γ***	***SYP22***	***ESD4***	***DBP1***	***DRM1***	***FLC***	***SOC1***	***LFY***	***AP1***
	AT1G04870	AT3G48430	AT1G01530	AT2G23080	AT4G15415	AT5G46860	AT4G15880	AT2G25620	AT5G15380	AT5G10140	AT2G45660	AT5G61850	AT1G69120
Aa-0	0	0	11	0	0	0	0	0	0	0	0	0	0
Br-0	0	0	4	0	0	0	0	0	0	0	0	0	0
Bs-1	0	0	7	0	0	0	0	0	0	0	0	0	0
Bsch-0	0	0	22	0	0	0	0	0	0	0	0	0	0
Ca-0	0	0	11	0	0	0	0	0	0	0	0	0	0
Col-0	0	0	6	0	0	0	0	0	0	0	0	0	0
Cvi-0	0	0	11	0	0	0	0	0	0	0	0	0	0
Ema-1	0	0	12	0	0	0	0	0	1	0	1	0	0
Gr-1	0	0	12	0	0	0	0	0	0	1	0	0	1
Gy-0	0	0	11	0	0	0	0	0	0	0	0	0	0
Hey-1	0	0	9	0	0	0	0	0	0	0	0	0	0
Hs-0	0	0	16	0	0	0	0	0	0	0	0	0	0
Kelsterbach-4	0	0	9	0	0	0	0	0	0	0	0	0	0
Kz-9	0	0	0	0	0	0	0	0	0	0	0	0	0
Ms-0	0	0	1	0	0	0	0	1	0	0	0	0	0
Nw-0	0	0	3	0	0	0	0	0	0	0	0	0	0
Per-1	0	0	0	0	0	0	0	0	0	0	0	0	0
Pro-0	0	0	5	0	1	0	0	0	0	0	0	0	0
Rd-0	0	0	0	0	0	0	0	0	0	0	0	0	0
Se-0	0	0	1	0	0	0	0	0	0	0	0	0	0
Sp-0	0	0	1	0	0	0	0	0	0	4	0	0	0
Stw-0	0	0	14	0	0	0	0	0	0	3	0	0	0
Ta-0	0	0	0	0	0	0	0	0	0	1	0	0	0
Tscha-1	0	0	5	0	0	0	0	0	0	0	0	0	0
Tu-0	0	0	8	0	0	0	0	0	0	1	0	0	0
Ty-0	0	0	0	0	0	0	0	0	0	0	0	0	0
Wa-1	0	0	17	0	0	0	0	0	0	0	0	0	0

Table S6. Correlation analysis between flowering time, leaf number and DNA methylation level of gene coding region of different *A. thaliana* accessions.

	FT	LN	*FVE*	*FY*	*FLD*	*PEP*	*HDA5*	*PRP39-1*	*FCA*	*FLK*	*FPA*	*LD*	*PCFS4*	*DRM2*	*HDA6*	*PRMT5*	*PRMT10*	*REF6*	*AGL28*	*CK2*	*PP2A-B'γ*	*SYP22*	*ESD4*	*DBP1*	*DRM1*	*FLC*	*SOC1*	*LFY*	*AP1*
FT			.016*	.039*	.007**	.000**	.006**	.027*	.116	.138	.080	.192	.193	.215	.089	.070	.868	.082	.053	.895	.061	.251	.326	.784	.712	.278	.353	.199	.517
LN			.016*	.032*	.007**	.000**	.006**	.027*	.123	.134	.078	.205	.197	.223	.103	.070	.867	.079	.050	.876	.065	.246	.326	.705	.714	.238	.350	.194	.521
FVE				.083	.000**	.001**	.001**	.007**	.008**	.001**	.000**	.191	.005**	.028*	.002**	.019*	.397	.004**	.778	.045*	.003**	.260	.609	.020*	.079	.660	.747	.790	.381
FY					.007**	.008**	.015*	.002**	.034*	.004**	.006**	.370	.010**	.465	.378	.022*	.005**	.049*	.903	.227	.076	.016*	.010**	.083	.235	.625	.689	.699	.518
FLD						.000**	.000**	.001**	.002**	.000**	.000**	.099	.001*8	.002**	.038*	.001**	.307	.000**	.790	.033*	.001**	.246	.031*	.006**	.306	.968	.433	.744	.792
PEP							.008**	.004**	.037*	.001**	.002**	.240	.010**	.258	.034*	.001**	.718	.006**	.078	.625	.011*	.049*	.182	.121	.308	.432	.529	.152	.764
HDA5								.000**	.007**	.011*	.006**	.071	.003**	.228	.001**	.309	.370	.002**	.438	.157	.001**	.246	.042*	.036*	.167	.831	.138	.085	.952
PRP39-1									.073	.003**	.001**	.473	.013**	.064	.040*	.004**	.008**	.002**	.650	.082	.000**	.173	.101	.029*	.708	.646	.571	.182	.868
FCA										.011*	.054	.000*8	.000**	.001**	.017*	.078	.614	.077	.853	.149	.000**	.047*	.009**	.532	.599	.039	.616	.680	.826
FLK											.000**	.157	.000**	.027*	.022*	.026*	.002**	.000**	.460	.004**	.000**	.083	.059	.005*	.280	.895	.807	.449	.131
FPA												.529	.013*	.015*	.021*	.067	.170	.004**	.965	.022*	.007**	.191	.176	.002**	.165	.574	.212	.780	.813
LD													.000**	.046*	.154	.439	.956	.196	.983	.634	.002**	.294	.254	.729	.656	.075	.979	.708	.360
PCFS4														.008**	.022*	.138	.129	.002**	.735	.043*	.000**	.047*	.017*	.039*	.506	.011	.804	.432	.863
DRM2															.162	.208	.682	.058	.827	.074	.000**	.648	.175	.117	.864	.385	.997	.459	.962
HDA6																.675	.671	.013*	.289	.204	.009**	.020*	.549	.627	.393	.767	.632	.618	.409

PRMT5	.235	.039*	.269	.723	.149	.112	.547	.519	.467	.688	.131	.138	.941
PRMT10		.028*	.906	.012*	.110	.454	.129	.308	.439	.924	.148	.036*	.069
REF6			.671	.025*	.004**	.052	.151	.012	.055	.665	.855	.101	.590
AGL28				.083	.501	.834	.663	.526	.739	.600	.476	.977	.149
CK2					.017*	.986	.091	.129	.714	.801	.454	.114	.977
PP2A-B'γ						.469	.013*	.077	.278	.528	.475	.155	.684
SYP22							.177	.961	.429	.889	.522	.112	.719
ESD4								.740	.924	.668	.865	.311	.264
DBP1									.331	.884	.181	.043*	.824
DRM1										.686	.264	.985	.660
FLC											.667	.847	.609
SOC1												.627	.639
LFY													.714
AP1													

FT: Flowering time; LN: Leaf number; * Correlation is significant at the 0.05 level (2-tailed); ** Correlation is also significant at the 0.01 level (2-tailed).

Table S7. Correlation analysis between flowering time, leaf number and CG methylation level of gene coding region of different *A. thaliana* accessions.

	FT	LN	*FVE*	*FY*	*FLD*	*PEP*	*HDA5*	*PRP39-1*	*FCA*	*FLK*	*FPA*	*LD*	*PCFS4*	*DRM2*	*HDA6*	*PRMT5*	*PRMT10*	*REF6*	*AGL28*	*CK2*	*PP2A-B'γ*	*SYP22*	*ESD4*	*DBP1*	*DRM1*	*FLC*	*SOC1*	*LFY*	*AP1*
FT			.021*	.033*	.006**	.000**	.007**	.023*	.133	.134	.083	.328	.186	.059	.089	.072	.868	.088	.131	.912	.060	.251	.295	.662	.747	.271	.389	.199	.357
LN			.021*	.027*	.006**	.000**	.007**	.022*	.140	.130	.082	.348	.190	.052	.102	.072	.867	.084	.138	.893	.063	.246	.296	.580	.750	.215	.368	.194	.387
FVE				.084	.000**	.001**	.001**	.006**	.010**	.000**	.000**	.095	.003**	.001**	.004**	.016*	.367	.003**	.524	.036*	.003**	.255	.596	.009**	.079	.484	.357	.785	.871
FY					.009**	.007**	.014*	.002**	.044*	.004**	.008**	.312	.012*	.061	.365	.024*	.006**	.046*	.792	.213	.090	.014*	.010**	.084	.169	.266	.556	.786	.652
FLD						.000**	.000**	.001**	.003**	.000**	.000**	.050*	.001**	.000**	.041*	.002**	.315	.000**	.193	.037*	.001**	.219	.027*	.007**	.328	.703	.239	.811	.996
PEP							.008**	.003**	.041*	.001**	.002**	.337	.009**	.015*	.034*	.002**	.718	.006**	.394	.634	.011*	.049*	.159	.099	.376	.459	.265	.152	.771
HDA5								.000**	.008**	.007**	.006**	.060	.003**	.028*	.001**	.327	.330	.001**	.504	.145	.001**	.225	.043*	.029*	.178	.357	.113	.076	.731
PRP39-1									.063	.003**	.002**	.332	.011*	.029*	.036*	.003**	.008**	.002**	.431	.086	.000**	.148	.099	.030*	.774	.796	.539	.186	.655
FCA										.013*	.071	.000**	.000**	.001**	.023*	.061	.625	.084	.550	.148	.000**	.052	.007**	.585	.562	.562	.547	.659	.661
FLK											.000**	.080	.000**	.000**	.021*	.028*	.003**	.000**	.801	.004**	.000**	.079	.063	.005**	.384	.459	.501	.440	.610
FPA												.263	.015*	.000**	.024*	.072	.174	.004**	.256	.021*	.007**	.188	.162	.002**	.192	.612	.115	.804	.741
LD													.000**	.008**	.055	.499	.802	.314	.763	.159	.000**	.241	.077	.689	.813	.851	.911	.675	.395
PCFS4														.002**	.025*	.124	.129	.002**	.923	.045*	.000**	.047*	.014*	.049*	.605	.393	.305	.435	.489
DRM2															.228	.029*	.450	.002**	.158	.002**	.001**	.884	.111	.003**	.460	.463	.631	.801	.196
HDA6																.722	.700	.012*	.519	.220	.009**	.021*	.612	.697	.420	.398	.625	.614	.581

PRMT5	.219	.040*	.226	.734	.138	.106	.478	.517	.514	.669	.362	.139	.834
PRMT10		.026*	.905	.011*	.106	.454	.152	.319	.358	.607	.578	.036	.158
REF6			.458	.026*	.005**	.051	.173	.015*	.097	.375	.580	.095	.932
AGL28				.498	.741	.668	.292	.480	.414	.981	.545	.625	.547
CK2					.020*	.991	.115	.121	.766	.186	.309	.111	.366
PP2A-B'γ						.474	.012*	.086	.281	.579	.639	.155	.995
SYP22							.169	.975	.566	.601	.866	.112	.960
ESD4								.737	.928	.538	.765	.362	.185
DBP1									.410	.530	.031*	.069	.271
DRM1										.769	.323	.990	.694
FLC											.694	.455	.624
SOC1												.698	.783
LFY													.788
AP1													

FT: Flowering time; LN: Leaf number; * Correlation is significant at the 0.05 level (2-tailed); ** Correlation is also significant at the 0.01 level (2-tailed).

Table S8. Correlation analysis between flowering time, leaf number and CHG methylation level of gene coding region of different *A. thaliana* accessions.

	FT	LN	*FVE*	*FY*	*FLD*	*PEP*	*HDA5*	*PRP39-1*	*FCA*	*FLK*	*FPA*	*LD*	*PCFS4*	*DRM2*	*HDA6*	*PRMT5*	*PRMT10*	*REF6*	*AGL28*	*CK2*	*PP2A-B'γ*	*SYP22*	*ESD4*	*DBP1*	*DRM1*	*FLC*	*SOC1*	*LFY*	*AP1*
**FT**			.766	.322	.694	NA	.092	.564	.237	.647	.169	.254	.514	.540	.728	.072	NA	.389	.236	.430	NA	NA	.578	.168	.263	.618	NA	NA	NA
**LN**			.765	.291	.640	NA	.110	.533	.266	.608	.180	.255	.505	.579	.675	.072	NA	.422	.207	.440	NA	NA	.595	.161	.268	.599	NA	NA	NA
FVE				.291	.453	NA	.171	.627	.871	.639	.171	.737	.033*	.675	.795	.182	NA	.645	.538	.795	NA	NA	.653	.604	.795	.758	NA	NA	NA
FY					.004**	NA	.824	.393	.346	.007**	.194	.854	.469	.020*	.155	.479	NA	.215	.537	.155	NA	NA	.847	.000**	.155	.562	NA	NA	NA
FLD							.129	.898	.276	.051	.004**	.739	.700	.026*	.007*8	.124	NA	.435	.278	.661	NA	NA	.446	.004**	.661	.602	NA	NA	NA
PEP							NA	NA	NA	NA	NA	NA	NA	NA	NA	NA	NA	NA	NA	NA	NA	NA	NA	NA	NA	NA	NA	NA	NA
HDA5								.818	.561	.583	.007**	.784	.660	.081	.704	.010**	NA	.247	.556	.086	NA	NA	.445	.448	.704	.653	NA	NA	NA
PRP39-1									.565	.936	.804	.787	.494	.283	.722	.724	NA	.482	.513	.394	NA	NA	.842	.465	.394	.442	NA	NA	NA
FCA										.575	.004**	.524	.060	.084	.924	.202	NA	.585	.661	.457	NA	NA	.615	.169	.000**	.888	NA	NA	NA
FLK											.492	.778	.887	.029*	.003**	.620	NA	.025*	.241	.003**	NA	NA	.552	.583	.731	.684	NA	NA	NA
FPA												.784	.191	.190	.704	.010**	NA	.827	.437	.704	NA	NA	.958	.081	.086	.653	NA	NA	NA
LD													.445	.283	.805	.722	NA	.916	.088	.980	NA	NA	.704	.911	.912	.790	NA	NA	NA
PCFS4														.797	.527	.531	NA	.862	.325	.527	NA	NA	.259	.660	.125	.453	NA	NA	NA
DRM2															.881	.905	NA	.235	.209	.152	NA	NA	.746	.013*	.464	.128	NA	NA	NA
HDA6																.783	NA	.736	.105	.849	NA	NA	.742	.704	.849	.821	NA	NA	NA

PRMT5	NA	.626	.930	.783	NA	NA	.635	.583	.783	.000	NA	NA	NA
PRMT10			NA	NA	NA	NA	NA	NA	NA	NA	NA	NA	NA
REF6			.997	.000**	NA	NA	.850	.827	.420	.960	NA	NA	NA
AGL28				.862	NA	NA	.853	.730	.211	.502	NA	NA	NA
CK2					NA	NA	.742	.704	.849	.821	NA	NA	NA
PP2A-B'γ						NA	NA	NA	NA	NA	NA	NA	NA
SYP22							NA	NA	NA	NA	NA	NA	NA
ESD4								.958	.139	.696	NA	NA	NA
DBP1									.086	.653	NA	NA	NA
DRM1										.821	NA	NA	NA
FLC											NA	NA	NA
SOC1												NA	NA
LFY													NA
AP1													

FT: Flowering time; LN: Leaf number; * Correlation is significant at the 0.05 level (2-tailed); ** Correlation is also significant at the 0.01 level (2-tailed); NA, not applicable.

Table S9. Correlation analysis between flowering time, leaf number and CHH methylation level of gene coding region of different *A. thaliana* accessions.

	FT	LN	*FVE*	*FY*	*FLD*	*PEP*	*HDA5*	*PRP39-1*	*FCA*	*FLK*	*FPA*	*LD*	*PCFS4*	*DRM2*	*HDA6*	*PRMT5*	*PRMT10*	*REF6*	*AGL28*	*CK2*	*PP2A-B'γ*	*SYP22*	*ESD4*	*DBP1*	*DRM1*	*FLC*	*SOC1*	*LFY*	*AP1*
**FT**			.527	NA	NA	NA	NA	NA	.603	NA	NA	NA	NA	.818	NA	.357	NA	NA	.147	NA	.158	NA	NA	.686	.728	.373	.728	NA	.684
**LN**			.541	NA	NA	NA	NA	NA	.635	NA	NA	NA	NA	.769	NA	.387	NA	NA	.138	NA	.161	NA	NA	.620	.762	.370	.762	NA	.757
FVE				NA	NA	NA	NA	NA	.783	NA	NA	NA	NA	.731	NA	.849	NA	NA	.838	NA	.849	NA	NA	.849	.849	.704	.849	NA	.849
FY					NA	NA	NA	NA	NA	NA	NA	NA	NA	NA	NA	NA	NA	NA	NA	NA	NA	NA	NA	NA	NA	NA	NA	NA	NA
FLD						NA	NA	NA	NA	NA	NA	NA	NA	NA	NA	NA	NA	NA	NA	NA	NA	NA	NA	NA	NA	NA	NA	NA	NA
PEP							NA	NA	NA	NA	NA	NA	NA	NA	NA	NA	NA	NA	NA	NA	NA	NA	NA	NA	NA	NA	NA	NA	NA
HDA5								NA	NA	NA	NA	NA	NA	NA	NA	NA	NA	NA	NA	NA	NA	NA	NA	NA	NA	NA	NA	NA	NA
PRP39-1									NA	NA	NA	NA	NA	NA	NA	NA	NA	NA	NA	NA	NA	NA	NA	NA	NA	NA	NA	NA	NA
FCA										NA	NA	NA	NA	.620	NA	.783	NA	NA	.377	NA	.783	NA	NA	.783	.783	.583	.783	NA	.783
FLK											NA	NA	NA	NA	NA	NA	NA	NA	NA	NA	NA	NA	NA	NA	NA	NA	NA	NA	NA
FPA												NA	NA	NA	NA	NA	NA	NA	NA	NA	NA	NA	NA	NA	NA	NA	NA	NA	NA
LD													NA	NA	NA	NA	NA	NA	NA	NA	NA	NA	NA	NA	NA	NA	NA	NA	NA
PCFS4														NA	NA	NA	NA	NA	NA	NA	NA	NA	NA	NA	NA	NA	NA	NA	NA
DRM2															NA	.731	NA	NA	.313	NA	.731	NA	NA	.731	.731	.946	.731	NA	NA
HDA6																NA	NA	NA	NA	NA	NA	NA	NA	NA	NA	NA	NA	NA	NA

PRMT5	NA	NA	.147	NA	.849	NA	NA	.849	.849	.704	.849	NA	.849
PRMT10		NA	NA	NA	NA	NA	NA	NA	NA	NA	NA	NA	NA
REF6			NA	NA	NA	NA	NA	NA	NA	NA	NA	NA	NA
AGL28				NA	.713	NA	NA	.304	.438	.831	.438	NA	.438
CK2					NA	NA	NA	NA	NA	NA	NA	NA	NA
PP2A-B'γ						NA	NA	.849	.849	.704	.849	NA	.849
SYP22							NA	NA	NA	NA	NA	NA	NA
ESD4								NA	NA	NA	NA	NA	NA
DBP1									.849	.704	.849	NA	.849
DRM1										.704	NA	NA	.849
FLC											.704	NA	.518
SOC1												NA	.849
LFY													NA
AP1													

FT: Flowering time; LN: Leaf number; NA, not applicable.

Table S10. Number of methylated sites of autonomous pathway gene promoter regions of different *A. thaliana* accessions.

**Population**	**Flowering time***	**Leaf number***	**Number of methylated sites**													
			FVE	***FY***	***FLD***	***PEP***	***HDA5***	***PRP39-1***	***FCA***	***FLK***	***FPA***	***LD***	***PCFS4***	***DRM2***	***HDA6***	***PRMT5***
			AT2G19520	AT5G13480	AT3G10390	AT4G26000	AT5G61060	AT1G04080	AT4G16280	AT3G04610	AT2G43410	AT4G02560	AT4G04885	AT5G14620	AT5G63110	AT4G31120
Aa-0	35±1.0	17.7±0.8	16	0	3	0	32	0	18	0	2	0	89	0	7	8
Br-0	45±0.0	22.6±1.0	19	0	1	0	26	0	62	0	3	3	46	0	6	6
Bs-1	29±0.0	14.8±0.7	18	6	3	0	23	0	16	0	2	0	64	1	2	3
Bsch-0	30±0.9	14.7±1.1	33	0	1	0	31	0	72	1	12	2	167	0	12	9
Ca-0	28±0.0	14.3±0.7	34	0	1	0	34	0	27	0	9	3	133	0	1	4
Col-0	27±2.0	13.7±1.0	100	0	2	0	33	0	43	0	8	5	98	0	3	11
Cvi-0	27±0.0	13.7±0.9	8	0	2	0	17	0	66	0	1	0	141	0	10	0
Ema-1	30±1.5	15.3±0.9	29	0	2	0	35	0	69	0	12	8	127	0	5	6
Gr-1	38±0.0	18.5±0.7	19	0	1	0	26	0	62	0	3	3	66	0	6	6
Gy-0	31±0.8	15.7±0.6	9	0	2	0	41	0	0	0	8	3	88	0	3	3
Hey-1	45±0.8	22.5±1.0	42	0	3	0	28	0	26	0	5	4	104	0	1	11
Hs-0	24±0.0	12.4±0.6	19	0	6	0	17	0	73	0	12	5	119	1	2	8
Kelsterbach-4	24±0.0	11.9±0.7	17	15	1	0	31	0	0	0	12	1	112	0	15	8
Kz-9	21±0.6	10.6±0.8	13	0	2	0	2	0	0	0	0	1	84	0	3	4
Ms-0	29.4±1.8	14.3±1.1	46	0	8	0	32	0	63	0	1	6	75	0	11	4
Nw-0	21±0.4	10.4±0.5	19	0	3	0	38	0	33	0	12	6	72	0	10	10
Per-1	45±2.2	22.5±1.3	16	0	3	0	20	0	24	0	7	2	66	0	3	1
Pro-0	19±0.7	9.7±0.9	19	0	3	0	42	0	23	0	29	4	127	0	5	0
Rd-0	21±0.0	10.7±0.8	0	0	2	0	25	0	29	0	1	1	22	0	1	2
Se-0	55.4±1.2	27.3±0.9	20	0	1	0	44	0	0	0	15	2	78	0	13	12
Sp-0	39±0.8	19.7±0.7	68	0	2	0	36	0	33	0	0	0	80	0	4	1
Stw-0	33.8±2.0	16.7±1.0	44	0	3	0	32	0	30	0	7	4	98	0	9	2
Ta-0	46.5±1.1	23.9±1.0	19	0	3	0	42	0	23	0	29	4	58	0	5	0
Tscha-1	54±2.3	26.7±0.9	50	0	2	0	44	0	54	0	0	3	85	0	6	10
Tu-0	42±2.3	20.9±1.1	81	4	1	0	33	0	31	0	19	4	95	0	1	5
Ty-0	45.4±1.8	22.6±1.1	61	2	0	0	37	0	0	0	6	4	73	0	12	18
Wa-1	24±0.6	12.1±0.3	28	0	1	0	29	0	27	0	10	3	112	0	7	8

Table S10 (continued). Number of methylated sites of autonomous pathway gene promoter regions of different *A. thaliana* accessions.

Population	Flowering time*	Leaf number*	Number of methylated sites												
			PRMT10	***AGL28***	***REF6***	***CK2***	***PP2A-B'γ***	***SYP22***	***ESD4***	***DBP1***	***DRM1***	***FLC***	***SOC1***	***LFY***	***AP1***
			AT1G04870	AT1G01530	AT3G48430	AT2G23080	AT4G15415	AT5G46860	AT4G15880	AT2G25620	AT5G15380	AT5G10140	AT2G45660	AT5G61850	AT1G69120
Aa-0	35±1.0	17.7±0.8	5	4	5	8	3	31	8	0	15	8	0	0	0
Br-0	45±0.0	22.6±1.0	2	3	1	2	0	38	13	0	7	0	0	1	0
Bs-1	29±0.0	14.8±0.7	14	2	11	3	4	156	6	0	28	10	0	0	0
Bsch-0	30±0.9	14.7±1.1	5	3	5	7	4	17	5	0	22	12	0	0	0
Ca-0	28±0.0	14.3±0.7	11	2	13	5	0	107	1	0	22	4	0	0	0
Col-0	27±2.0	13.7±1.0	5	2	4	13	2	168	0	0	20	1	0	0	0
Cvi-0	27±0.0	13.7±0.9	0	2	2	3	4	0	32	0	0	4	0	0	0
Ema-1	30±1.5	15.3±0.9	10	4	2	10	3	27	4	0	13	3	0	0	0
Gr-1	38±0.0	18.5±0.7	2	3	1	2	0	38	13	0	7	0	0	1	0
Gy-0	31±0.8	15.7±0.6	11	2	6	9	0	80	4	0	25	0	0	0	0
Hey-1	45±0.8	22.5±1.0	0	5	9	8	4	43	11	2	21	11	1	0	0
Hs-0	24±0.0	12.4±0.6	0	1	4	10	4	39	3	0	18	12	0	0	0
Kelsterbach-4	24±0.0	11.9±0.7	0	3	0	9	3	164	3	0	10	9	0	0	0
Kz-9	21±0.6	10.6±0.8	8	3	7	4	2	80	2	0	25	0	0	0	0
Ms-0	29.4±1.8	14.3±1.1	0	4	9	8	0	55	1	0	0	0	0	0	0
Nw-0	21±0.4	10.4±0.5	0	2	4	2	7	63	0	0	4	4	0	0	0
Per-1	45±2.2	22.5±1.3	4	3	6	6	0	48	5	0	0	7	0	0	0
Pro-0	19±0.7	9.7±0.9	9	5	5	7	0	27	7	0	22	4	0	0	0
Rd-0	21±0.0	10.7±0.8	0	0	6	2	0	3	0	0	1	0	0	0	0
Se-0	55.4±1.2	27.3±0.9	9	0	6	10	4	68	0	0	13	0	0	0	0
Sp-0	39±0.8	19.7±0.7	5	5	4	7	0	42	12	0	0	5	0	0	0
Stw-0	33.8±2.0	16.7±1.0	6	4	13	12	3	44	8	2	24	11	0	0	0
Ta-0	46.5±1.1	23.9±1.0	9	5	5	7	0	27	7	0	22	4	0	0	0
Tscha-1	54±2.3	26.7±0.9	2	2	3	2	0	161	3	0	16	5	0	0	0
Tu-0	42±2.3	20.9±1.1	3	4	2	12	2	196	0	0	10	5	0	0	0
Ty-0	45.4±1.8	22.6±1.1	0	5	10	14	6	120	1	0	5	0	0	0	0
Wa-1	24±0.6	12.1±0.3	8	3	8	5	0	181	0	0	17	12	0	0	0

* Data are shown as mean ± standard errors

Table S11. Correlation analysis between flowering time, leaf number and methylation level of promoter region of different *A. thaliana* accessions.

	FT	LN	*FVE*	*FY*	*FLD*	*PEP*	*HDA5*	*PRP39-1*	*FCA*	*FLK*	*FPA*	*LD*	*PCFS4*	*DRM2*	*HDA6*	*PRMT5*	*PRMT10*	*REF6*	*AGL28*	*CK2*	*PP2A-B'γ*	*SYP22*	*ESD4*	*DBP1*	*DRM1*	*FLC*	*SOC1*	*LFY*	*AP1*
FT			.193	.496	.262	NA	.054	NA	.732	.728	.921	.876	.117	.324	.711	.160	.764	.404	.806	.477	.711	.764	.707	.434	.517	.530	.280	.282	NA
LN			.203	.479	.265	NA	.056	NA	.710	.675	.973	.864	.114	.361	.815	.188	.837	.387	.812	.471	.683	.785	.681	.451	.560	.543	.277	.307	NA
FVE				.922	.697	NA	.136	NA	.617	.946	.981	.119	.566	.434	.682	.077	.547	.086	.739	.002**	.880	.008**	.240	.480	.938	.923	.656	.453	NA
FY					.348	NA	.893	NA	.073	.751	.670	.202	.830	.357	.124	.639	.648	.933	.318	.482	.324	.008**	.550	.647	.995	.235	.751	.647	NA
FLD						NA	.483	NA	.119	.430	.828	.114	.752	.045*	.626	.176	.397	.809	.362	.859	.814	.146	.847	.538	.811	.484	.670	.252	NA
PEP							NA	NA	NA	NA	NA	NA	NA	NA	NA	NA	NA	NA	NA	NA	NA	NA	NA	NA	NA	NA	NA	NA	NA
HDA5								NA	.497	.978	.017*	.097	.984	.097	.196	.273	.381	.377	.955	.144	.728	.491	.182	.911	.814	.510	.775	.474	NA
PRP39-1									NA	NA	NA	NA	NA	NA	NA	NA	NA	NA	NA	NA	NA	NA	NA	NA	NA	NA	NA	NA	NA
FCA										.103	.489	.074	.152	.511	.911	.666	.090	.779	.070	.276	.710	.087	.080	.745	.224	.593	.758	.081	NA
FLK											.644	.627	.012*	.783	.145	.489	.952	1.000	.867	.984	.356	.328	.939	.783	.359	.096	.849	.783	NA
FPA												.050*	.201	.809	.826	.620	.141	.254	.532	.089	.976	.945	.239	.672	.124	.578	.675	.329	NA
LD													.559	.726	.928	.197	.677	.326	1.000	.064	.596	.942	.060	.482	.830	.520	.627	1.000	NA
PCFS4														.989	.343	.753	.494	.530	.953	.175	.228	.946	.371	.677	.089	.013	.701	.095	NA
DRM2															.155	.891	.448	.128	.435	.868	.179	.585	.829	.692	.133	.036	.783	.692	NA
HDA6																.059	.157	.848	.497	.462	.037*	.847	.725	.720	.163	.999	.221	.990	NA

PRMT5	.108	.675	.998	.083	.002**	.061	.048*	.853	.965	.963	.250	.981	NA
PRMT10		1.000	.068	.841	.253	.489	.371	.560	.000**	.919	.267	.356	NA
REF6			.706	.111	.708	.729	.374	.128	.896	.485	.161	1.000	NA
AGL28				.410	.712	.705	.184	.020*	.049	.339	.332	.053	NA
CK2					.160	.280	.119	.223	.389	.653	.771	.045	NA
PP2A-B'γ						.978	.963	.320	.887	.194	.356	.162	NA
SYP22							.011*	.446	.261	.582	.592	.369	NA
ESD4								.396	.269	.781	.418	.104	NA
DBP1									.156	.036*	.000**	.692	NA
DRM1										.079	.419	.298	NA
FLC											.156	.104	NA
SOC1												.783	NA
LFY													NA
AP1													

FT: Flowering time; LN: Leaf number; * Correlation is significant at the 0.05 level (2-tailed); ** Correlation is also significant at the 0.01 level (2-tailed); NA, not applicable.

Table S12. Correlation analysis between DNA methylation level of gene coding region and gene expression level among 15 *A. thaliana* accessions.

Accession	mC		mCG		mCHG		mCHH	
	P value	R^2	*P* value	R^2	*P* value	R^2	*P* value	R^2
FVE	0.03	0.32*	0.03	0.3*	0.87	0	0.87	0
FY	0.00	0.51**	0.00	0.51**	0.87	0	NA	NA
FLD	0.00	0.61**	0.00	0.61**	0.80	0.01	NA	NA
PEP	0.04	0.29*	0.04	0.29*	NA	NA	NA	NA
HDA5	0.03	0.33*	0.02	0.33*	0.97	0	NA	NA
PRP39-1	0.00	0.47**	0.01	0.45*	0.51	0.03	NA	NA
FCA	0.09	0.2	0.08	0.22	0.18	0.06	0.79	0.01
FLK	0.47	0.04	0.44	0.04	0.38	0	NA	NA
FPA	0.06	0.24	0.06	0.24	NA	NA	NA	NA
LD	0.48	0.04	0.34	0.07	0.93	0	NA	NA
PCFS4	0.14	0.15	0.14	0.15	0.61	0.06	NA	NA
DRM2	0.59	0.02	0.04	0.29	0.7	0.01	0.6	0.02
HDA6	0.28	0.09	0.27	0.09	0.81	0	NA	NA
PRMT5	0.08	0.2	0.08	0.21	NA	NA	0.46	0.04
PRMT10	0.28	0.09	0.28	0.09	NA	NA	NA	NA
REF6	0.17	0.13	0.16	0.14	0.52	0.03	NA	NA
AGL28	0.76	0.01	0.22	0.11	0.94	0	0.87	0
CK2	0.37	0.06	0.37	0.06	NA	NA	NA	NA
PP2A.B.γ	0.10	0.19	0.10	0.19	NA	NA	NA	NA
SYP22	0.28	0.09	0.28	0.09	NA	NA	NA	NA
ESD4	0.10	0.2	0.10	0.2	0.74	0.01	NA	NA
DBP1	0.83	0	0.81	0	0.90	0	NA	NA
DRM1	0.79	0.01	0.79	0.01	NA	NA	NA	NA
FLC	0.50	0.04	0.41	0.05	0.83	0	0.83	0
SOC1	0.20	0.12	0.20	0.12	NA	NA	NA	NA
LFY	0.93	0	0.93	0	NA	NA	NA	NA
AP1	0.92	0	0.92	0	NA	NA	NA	NA

mC, overall methylation level of gene coding region; mCG, methylation level in the CG context; mCHG, methylation level in the CHG context; mCHH, methylation level in the CHH context; * Correlation is significant at the 0.05 level (2-tailed); ** Correlation is also significant at the 0.01 level (2-tailed); NA, not applicable.

Table S13. Number of methylated sites of gene coding regions and the percentage decreases in methylation level of the six genes following 5-azac treatment in the Col-0, Br-0 and Tscha-1 accessions.

		FVE		***FY***		***FLD***		***PEP***		***HDA5***		***PRP39-1***	
	**Population**	**Number of methylated sites**	**Drop percentage (%)**	**Number of methylated sites**	**Drop percentage (%)**	**Number of methylated sites**	**Drop percentage (%)**	**Number of methylated sites**	**Drop percentage (%)**	**Number of methylated sites**	**Drop percentage (%)**	**Number of methylated sites**	**Drop percentage (%)**
**mC**	Col-0	10	50.0	36	26.5	58	15.9	20	20.0	36	25.0	50	25.4
		12	40.0	38	22.5	57	17.4	18	28.0	36	25.0	50	25.4
		13	35.0	40	18.4	59	14.5	19	24.0	37	22.9	52	22.4
	Br-0	20	51.2	42	28.8	57	17.4	25	24.2	46	25.8	55	40.2
		22	46.3	40	32.2	56	18.8	26	21.2	43	30.7	57	38.0
		25	39.0	40	32.2	54	21.7	25	24.2	48	22.6	60	34.8
	Tscha-1	27	54.2	36	40.0	59	24.4	27	41.3	42	40.0	55	40.9
		27	54.2	39	35.0	57	26.9	30	34.8	46	34.3	59	36.6
		26	55.9	38	36.7	61	21.8	34	26.1	50	28.6	63	32.3
**mCG**	Col-0	7	58.8	36	25.0	58	15.9	20	20.0	36	25.0	50	25.4
		11	35.3	38	20.8	57	17.4	18	28.0	36	25.0	50	25.4
		11	35.3	39	18.8	59	14.5	19	24.0	37	22.9	52	22.4
	Br-0	20	51.2	42	28.8	57	17.4	25	24.2	46	25.8	55	40.2
		22	46.3	40	32.2	56	18.8	26	21.2	43	30.7	57	38.0
		25	39.0	40	32.2	54	21.7	25	24.2	48	22.6	60	34.8
	Tscha-1	27	54.2	36	40.0	59	22.4	27	41.3	42	38.2	55	40.9
		26	55.9	39	35.0	57	25.0	30	34.8	46	32.4	59	36.6
		26	55.9	38	36.7	60	21.1	34	26.1	49	27.9	63	32.3

mC, Total number of methylated sites of gene coding regions; mCG, number of methylated sites in the CG context of gene coding regions.

Table S14. Methylated sites of the *FVE* and *FVE(CS)* coding regions in Col-0, *fve-3+pFVE::FVE* and *fve-3+pFVE::FVE(CS)*.

Population	methylated sites	Number			
		CG	CHG	CHH	Total
Col-0	74, 261, 433, 451, 552, 598, 628, 655, 692, 711, 905, 965, 983, 989, 1047, 1113, 1130, 1273, 1355, 1493	17	2	1	20
fve-3+pFVE::FVE(CS)	65, 68, 88, 136, 208, 295, 325, **433**, 523, 551, 560, **598**, **655**, 710, 728, 748, 788, 821, 842, 853, 881, 916, **983**, 1018, 1087, 1099, 1112, **1273**, 1429, 1454	7	18	5	30
fve-3+pFVE::FVE	**74**, 119, 121, 125, 208, 325, 371, **433**, **598**, **628**, **655**, 665, 676, 699, 710, **711**, 728, 764, 780, 788, 805, 842, 853, **905**, 908, 924, 927, 944, 949, **965**, **983**, **989**, 1013, 1018, **1047**, 1060, 1077, 1086, 1087, 1099, **1113**, **1130**, 1155, 1160, 1177, 1187, 1257, **1273**, **1355**, 1374, 1382, 1389, 1400, 1420, 1429, 1454, **1493**	20	27	10	57

The black bold numbers represent the same methylated sites of *FVE* and *FVE(CS)* in *fve-3+pFVE::FVE* and *fve-3+pFVE::FVE(CS)*, respectively, compared with Col-0.

Table S15. RT-qPCR primers.

Primer name	Primer sequence
FVE-Fq	5’-CTCGTCTGGCCTTCACTCTC-3’
FVE-Rq	5’-CTGTGGGAGTTCCCTGATTC-3’
FLD-Fq	5’-GCTTACCGAGGAAGAGATTG-3’
FLD-Rq	5’-TGCTTGACTGAGCAGGAAAC-3’
FY-Fq	5’-TACACCAGCACCGTTGTTAG-3’
FY-Rq	5’-GCAGCAAAGCTTGTTGAAGG-3’
PEP-Fq	5’-ACCTTATACCGGCGGGATTC-3’
PEP-Rq	5’-CCGGAACGATCATACGAAAC-3’
HDA5-Fq	5’-TCCGGAAGCATACCCATTCG-3’
HDA5-Rq	5’-AGCCTCTTGCGCACCTAAAC-3’
PRP39-1-Fq	5’-CCGCACTTCCACTTGCTTCC-3’
PRP39-1-Rq	5’-GGAGGGTCCCTCTGTATGTG-3’
FLC-Fq	5’-GTAGCCGACAAGTCACCTTC-3’
FLC-Rq	5’-CCGGAGGAGAAGCTGTAGAG-3’
SOC1-Fq	5’-ATTCGCCAGCTCCAATATGC-3’
SOC1-Rq	5’-TCAATCTGTTGCAGCTCCTC-3’
LFY-Fq	5’-CGAGCACGCTTGTGGGTATG-3’
LFY-Rq	5’-GGCGGAGAGTAGCAAATGAC-3’
AP1-Fq	5’-TTCACTCGCTCACTGAGTTC-3’
AP1-Rq	5’-GAAGACACAGTCGTGAGAAC-3’
ACTIN-Fq	5’-GGCTCCTCTTAACCCAAAGG-3’
ACTIN-Rq	5’-CAGTAAGGTCACGTCCAGCA-3’

Table S16. Primers used for *FVE* and *FVE(CS)* plant expression vector construction.

**Primer name**	**Primer sequence**	**Primer usage**
pFVE-F	5'-AGTACTCCGTCGAGCAAGTTAATACC-3'	pFVE-FVE
FVE-R	5'-TCTAGATAAGGCTTGGAGGCACAAGTC-3'	amplification
FVE(CS)-F	5'-ATGGAGAGTGATGAAGCAGCAGCAGTG-3'	FVE(CS)
FVE(CS)-R	5'-TTATGGCTTAGATGCACATGTC-3'	amplification
pFVE-F	5'-CCGTCGAGCAAGTTAATACC-3'	pFVE
pFVE-R	5'-TTTCCTCTCTCTCTTTTTAATTTTTC-3'	amplification
pFVE-F	5'-AGTACTCCGTCGAGCAAGTTAATACC-3'	
pFVE-R3	5'-TTCATCACTCTCCATTTTCCTCTCTCTCTT-3'	Overlap of
FVE(CS)-F1	5'-AAGAGAGAGAGGAAAATGGAGAGTGATGAA-3'	pFVE-FVE(CS)
FVE(CS)-R	5'-TCTAGATTATGGCTTAGATGCACATGTC-3'	

Table S17. Primers used for *FVE* and *FVE(CS)* methylation determination

**Primer name**	**Primer sequence**
FVE-Me-F1	5’-ATGGAGAGYGAYGAAGTAGTAG-3’
FVE-Me-R1	5’-TAACAACTTCACAATTTACTATAACC-3’
FVE-Me-F2	5’-TTTAGAGTAAATTGATGGAAGTGTG-3’
FVE-Me-R2	5’-AAACAACTAACTTATCCTTACCTC-3’
FVE-Me-F3	5’-GGGTATTAAGATAATGTTGAATTTG-3’
FVE-Me-R3	5’-AAATTTATACCAATTCTCRCATCCC-3’
FVE-Me-F4	5’-GTATTTAGTTYGAYGAGTGTATAAG-3’
FVE-Me-R4	5’-TTAACTACACRATCAAACTTCTTAC-3’
FVE-Me-F5	5’-TTTGAATATTTGGGATTATGATAGG-3’
FVE-Me-R5	5’-CACAAATCATAACATACRACTTAAAC-3’
FVE(CS)-Me-F1	5’-ATGGAGAGTGATGAAGTAGTAGTAG-3’
FVE(CS)-Me-R1	5’-TAACAACTTCACAATTAACTATAAC-3’
FVE(CS)-Me-F2	5’-TTTAGAGTAAATTGATGGAAGTGTG-3’
FVE(CS)-Me-R2	5’-AAACAACTAACTTATCCTTTCCTCC-3’
FVE(CS)-Me-F3	5’-GGGTATTAAGATAATGTTGAATTTG-3’
FVE(CS)-Me-R3	5’-AAATTAATACCAATTCTAACATCCC-3’
FVE(CS)-Me-F4	5’-ATATAGTTGAAGATGTGGTATTTAG-3’
FVE(CS)-Me-R4	5’-ACTTCTTACTAACCCTATCATAATC-3’
FVE(CS)-Me-F5	5’-TATGTTGGTTATAGGGATAAAGTTG-3’
FVE(CS)-Me-R5	5’-TATAACTTAAATACACATATCATTAC-3’
FY-Me-F1	5’-ATGTAYGTYGGYGGYGATATGCA-3’
FY-Me-R1	5’-ATTAATCATATRCCTAAAAAATC-3’
FY-Me-F2	5’-GGTATATGATTAATTTATTAGG-3’
FY-Me-R2	5’-CCCACAAAAAAATTAAAGATCC-3’
FY-Me-F3	5’-TGGGTATAYGAAAGATGTAATA-3’
FY-Me-R3	5’-CCAAAAAGRGATAAATAAAAACC-3’
FY-Me-F4	5’-GTTTTTTGGAAGTGGTTAGTAG-3’
FLD-Me-F1	5’-ACTRCCTACTAATATTACTAATTA-3’
FLD-Me-R1	5’-AACTTATTCAATRCCTCCAAC-3’
FLD-Me-F2	5’-GAGATTGAATTYGGTGTGGTGTT-3’
FLD-Me-R2	5’-CCTTATAAAAAAAAAAACCAARC-3’
FLD-Me-F3	5’-GGATTATAGYGAGATAGTTTGG-3’
FLD-Me-R3	5’-GTACACARCACCATATCACCCTC-3’
FLD-Me-F4	5’-TGGAAATAAAGTGTATGAGGGT-3’
FLD-Me-R4	5’-CTRCTAATATATCATAATCATCRC-3’
FLD-Me-F5	5’-GTAGTGGGAGCTTTAGGYGATG-3’
FLD-Me-R5	5’-CATCACCACCTCTAACTTCTCTC-3’
FLD-Me-F6	5’-GGTTTTTGATTTGAGAGAAGTTAG-3’
FLD-Me-R6	5’-CAAAAATTCTCCAAAAATCATT-3’
PEP-Me-F1	5’-ATGGTYGTYGTYGTAGATTTYG-3’
PEP-Me-R1	5’-AATTCACAACTTTAATAATCAC-3’

PEP-Me-F2	5’-GTTGTGAATTTTTATTTTTTAG-3’
PEP-Me-R2	5’-AAAACTARCAACAAAAACACCA-3’
PEP-Me-F3	5’-TTGTTAGAGGAAATATYGTTTT-3’
PEP-Me-R3	5’-CTCATTTTCAAAARCTAATATT-3’
PEP-Me-F4	5’-GGTAGATTTTTTAAATATAAT-3’
PEP-Me-R4	5’-ATAATAAACTCCTACACACACA-3’
PEP-Me-F5	5’-GTGTAGGAGTTTATTATTAATTA-3’
PEP-Me-R5	5’-CAAATTATTATTATAAAACRTT-3’
HDA5-Me-F1	5’-ATGGTTATGGTYGGAGAATTT-3’
HDA5-Me-R1	5’-CCCATARCTTCATCTRCCTCAA-3’
HDA5-Me-F2	5’-GGGTATTATGTTGAGGTAGATG-3’
HDA5-Me-R2	5’-CARCACTATCTTTCCTTATRCA-3’
HDA5-Me-F3	5’-GATAGTGTTGGYGTTAGAGGGA-3’
HDA5-Me-R3	5’-AACCAACACATACCTTTTTCAATC-3’
HDA5-Me-F4	5’-GGATAGGTYGATGGATTGAAAA-3’
HDA5-Me-R4	5’-ARCCTCARCTTCTTCTTTAAAT-3’
PRP39-1-Me-F1	5’-ATGTTTTTATTAGGAAGGTTTT-3’
PRP39-1-Me-R1	5’-CRCACATAAAAATAAAACCTCC-3’
PRP39-1-Me-F2	5’-TGGTTATAAGGAGGTTTTATTT-3’
PRP39-1-Me-R2	5’-TATACATAATCAAATRCTTCAAC-3’
PRP39-1-Me-F3	5’-GATGTTGAGAAAGTTAGGAGGA-3’
PRP39-1-Me-R3	5’-CAAAARCARCTTATRCTAATARCC-3’
PRP39-1-Me-F4	5’-GGTGGTTATGTTGYGTAGTTTT-3’
PRP39-1-Me-R4	5’-AACAACCARCARCAATAATAARC-3’

Note: Y=T/C, R=A/G

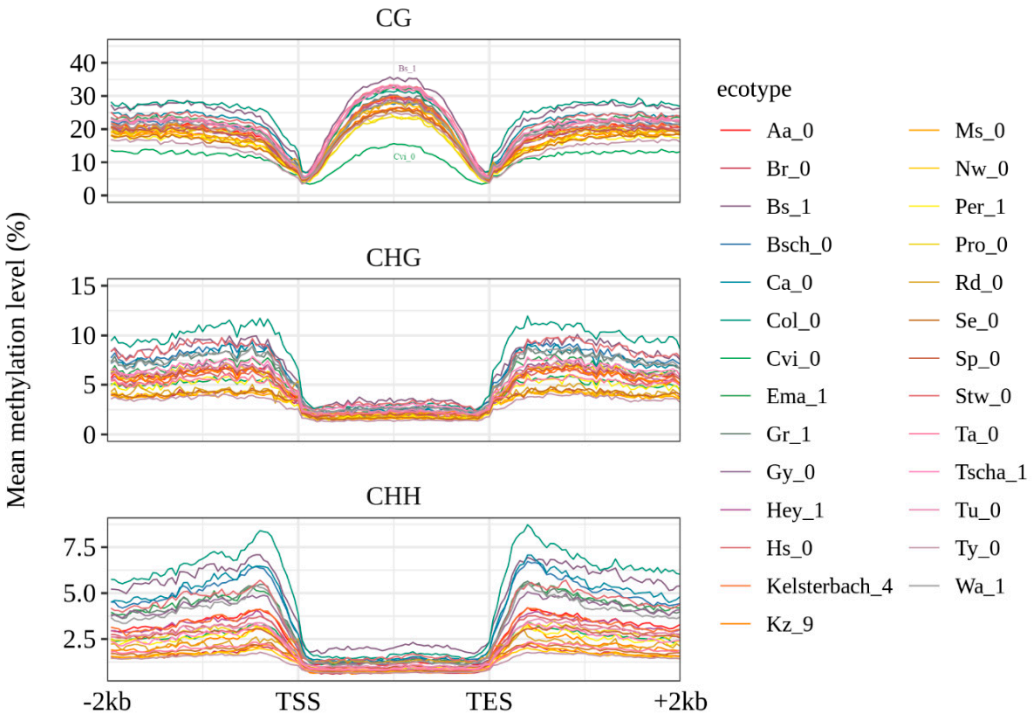

Figure S1. Distribution of DNA methylation level of protein-coding genes, including a 2kb window upstream of the transcriptional start site (TSS) and downstream of the transcription end site (TES).

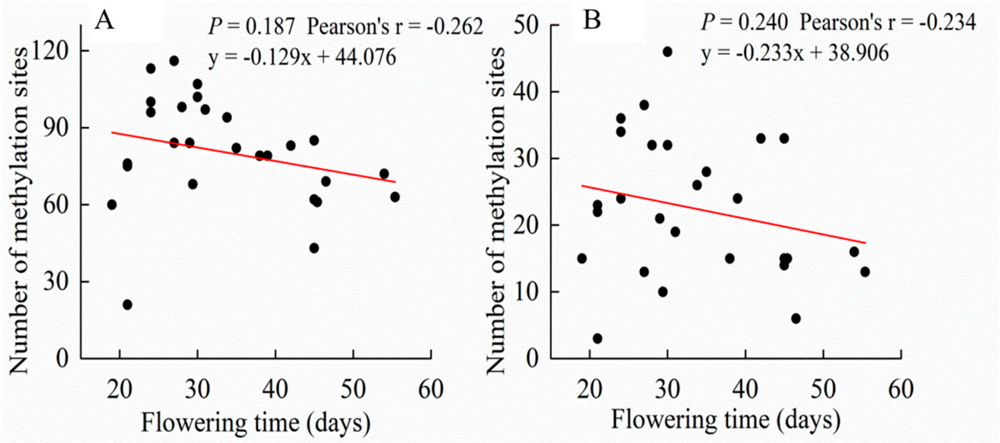

Figure S2. Correlation plot of methylation levels of the coding regions of *FWA* gene (A) and the promoter region (B).

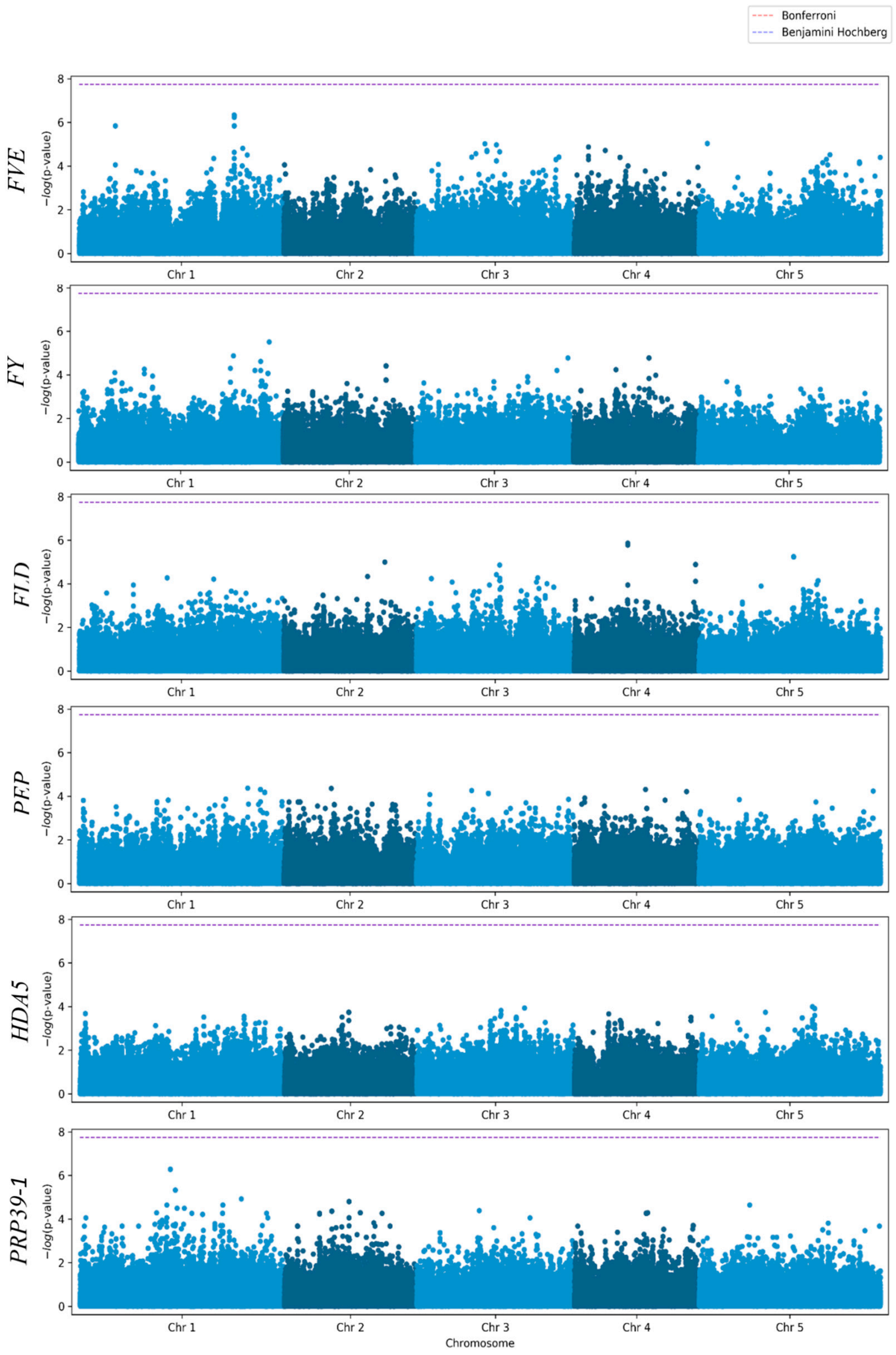

Figure S3. GWAS for CG methylation level of *FVE*, *FY*, *FLD*, *PEP*, *HDA5* and *PRP 39-1* genes. Horizontal dash lines represent 5% significance thresholds corrected for multiple testing using bonferroni.

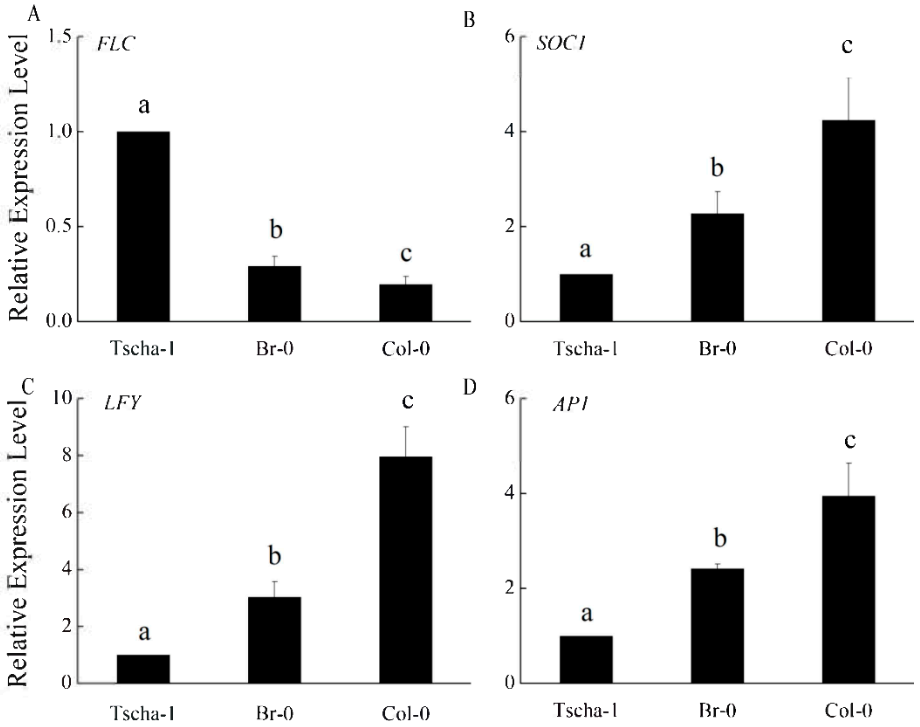

Figure S4. Relative expression levels of the downstream genes *FLC* (A), *SOC1* (B), *LFY* (C) and *AP1* (D) in the autonomous pathway among Col-0, Br-0 and Tscha-1 (Different letters indicate significant differences among the different accessions at the level of $P < 0.05$).

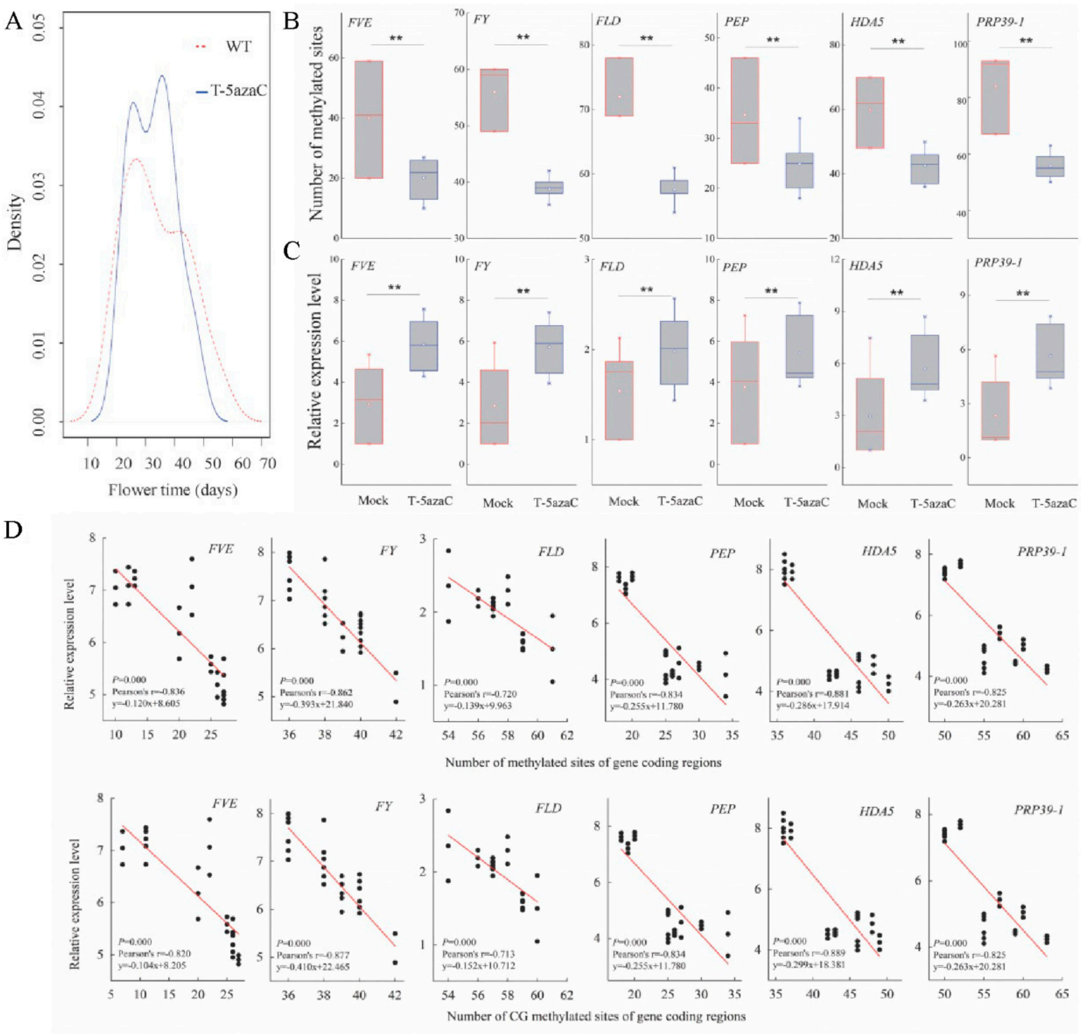

Figure S5. Flowering time, numbers of DNA methylation sites, relative expression levels and correlation analysis of the *FVE*, *FY*, *FLD*, *PEP*, *HDA5* and *PRP39-1* genes among Col-0, Br-0 and Tscha-1 accessions treated with or without 5-azac. A, Flowering time of 27 *A. thaliana* accessions treated (T-5azaC) or not treated (WT) with 5-azac. Y-axis: ratio of the number of individuals to the total number of individuals per day among all 27 *A. thali*ana accessions; B, number of methylated sites of the six genes among Col-0, Br-0 and Tscha-1 accessions treated with (T-5azaC) or without 5-azac (Mock); C, relative expression levels of the six genes among Col-0, Br-0 and Tscha-1 accessions treated with (T-5azaC) or without 5-azac (Mock). Mock: Col-0, Br-0 and Tscha-1 accessions not treated with 5-azac; T-5azaC: Col-0, Br-0 and Tscha-1 accessions treated with 50 μM 5-azac. ** Correlation is significant at the level of $P < 0.01$; D, correlation analysis of relative expression level and number of methylated sites of the six genes among Col-0, Br-0 and Tscha-1 accessions after 5-azac treatment.

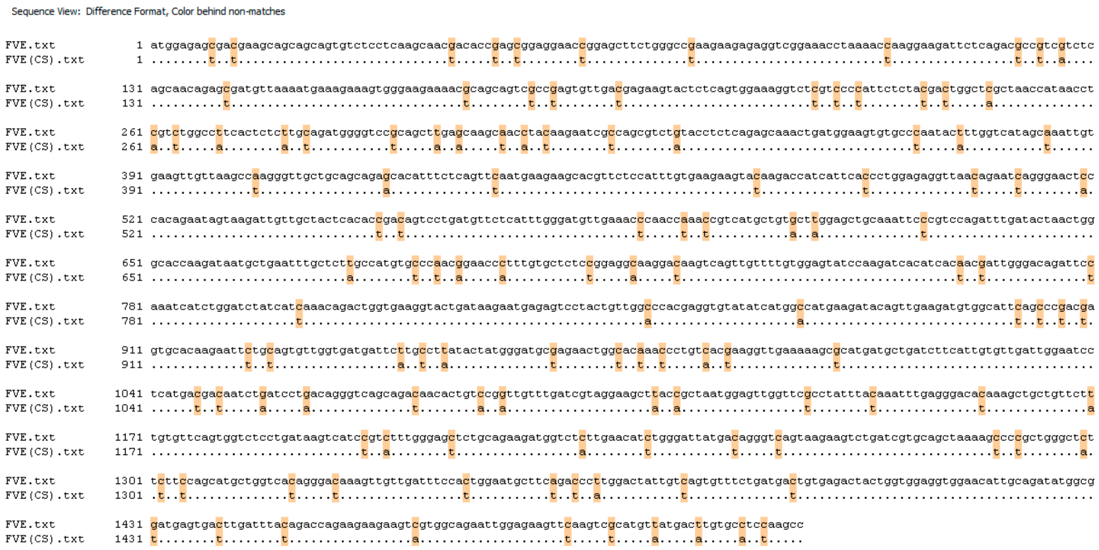

Figure S6. Alignment of the *FVE* and *FVE(CS)* gene sequences.
